# Small-diameter artery grafts engineered from pluripotent stem cells maintain 100% patency in an allogeneic rhesus macaque model

**DOI:** 10.1016/j.xcrm.2025.102002

**Published:** 2025-03-10

**Authors:** Jue Zhang, Diana Marcela Tabima, David Vereide, Weifeng Zeng, Nicholas J. Albano, Sarah Lyon, Peter J. Nicksic, Ellen C. Shaffrey, Robert E. George, Mitchell D. Probasco, Elizabeth S. Perrin, Yiyang Xu, Matthew E. Brown, Ron Stewart, Naomi C. Chesler, Lih-Sheng Turng, Samuel O. Poore, Igor I. Slukvin, James A. Thomson, John P. Maufort

**Affiliations:** 1 Morgridge Institute for Research, Madison, WI 53715, USA; 2 Wisconsin National Primate Research Center, University of Wisconsin–Madison, Madison, WI 53715, USA; 3 School of Medicine and Public Health, Division of Plastic and Reconstructive Surgery, University of Wisconsin–Madison, Madison, WI 53792, USA; 4 School of Medicine and Public Health, Department of Surgery, University of Wisconsin–Madison, Madison, WI 53792, USA; 5 Wisconsin Institute for Discovery, University of Wisconsin–Madison, Madison, WI 53715, USA; 6 Department of Mechanical Engineering, University of Wisconsin–Madison, Madison, WI 53706, USA; 7 Department of Biomedical Engineering, University of Wisconsin–Madison, Madison, WI 53706, USA; 8 Edwards Lifesciences Foundation Cardiovascular Innovation and Research Center, University of California Irvine, Irvine, CA 92617, USA; 9 Department of Cell & Regenerative Biology, University of Wisconsin–Madison, Madison, WI 53706, USA; 10 Department of Pathology and Laboratory Medicine, University of Wisconsin–Madison, Madison, WI 53705, USA

**Keywords:** pluripotent stem cells, endothelial cells, ePTFE vascular grafts, vascular disease, peripheral bypass, rhesus macaque, small-diameter vascular grafts, issue-engineering, artery, allogeneic

## Abstract

Autologous vascular grafts, the only clinically approved option for small-diameter (<6 mm) revascularizations, require invasive harvesting and have limited availability and variable quality. To address these challenges, we develop a 3-mm-diameter artery graft by using arterial endothelial cells (AECs) derived from pluripotent stem cells (PSCs). After establishing technologies for pure AEC generation and expanded polytetrafluoroethylene (ePTFE) graft coating, we engineer artery grafts by seeding the inner lumen of ePTFE vascular grafts with either major histocompatibility complex (MHC) mismatched unmodified-wild-type (MHC-WT) AECs or MHC class I/II double knockout (MHC-DKO) AECs. Their function is evaluated in a rhesus arterial interposition grafting model. MHC-WT grafts maintained 100% patency for 6 months, significantly better than naked and MHC-DKO grafts. Additionally, the endothelium of MHC-WT grafts is repopulated with host cells, supporting long-term patency. Collectively, our study demonstrates that PSC-derived MHC-WT artery grafts provide an unlimited homogenous resource for allogeneic arterial revascularization.

## Highlights

• A 3-mm-diameter graft is developed using PSC-derived arterial endothelial cells
• MHC-WT grafts maintain 100% patency for 6 months in an allogeneic rhesus model
• The graft endothelium is repopulated with host cells, ensuring the long-term patency

## Graphical abstract

**Figure:**
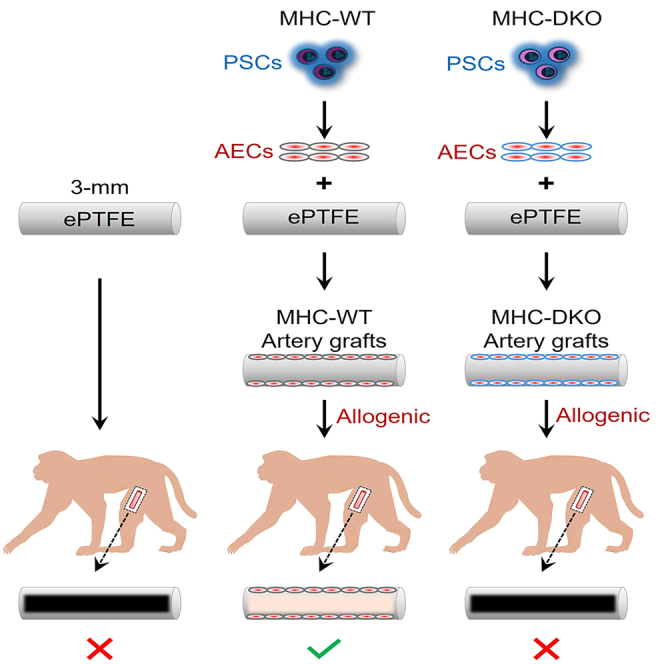

## Teaser

Jue Zhang and colleagues engineer PSC-derived artery grafts for small-diameter revascularization. MHC-WT grafts show 100% patency in a rhesus model, outperforming MHC-DKO and naked grafts. Host cell repopulation supports long-term patency. The grafts offer an unlimited resource for allogeneic arterial revascularization.

## Introduction

Occlusive arterial disease remains the leading cause of death globally.^1^^,^^2^ With respect to ischemic cardiomyopathy, the Surgical Treatment for Ischemic Heart Failure (STITCH) trial demonstrated that surgical revascularization improves patient prognosis and mortality at long-term follow-up while there does not appear to be such a benefit from percutaneous coronary intervention.^3^^,^^4^ As a result, surgical revascularization with vascular bypass surgery is the standard of care for the most severe forms of stable ischemic heart disease. According to the 2023 update from the American Heart Association, 291,585 inpatient coronary artery bypass procedures and 60,335 inpatient peripheral arterial bypass procedures were performed in the United States in 2018.^5^ Vascular bypass surgery primarily relies on autologous vessels, with the left internal mammary artery (IMA) being the most common choice for coronary bypass.^6^ While the IMA is efficacious, autologous grafts require invasive harvesting and some patients lack suitable vessels for harvest.^7^ Furthermore, surgeons operating on patients who have already undergone a bypass with the left IMA may benefit from the increased array of graft options afforded by synthetic grafts. Allogenic vessels are an alternative but are limited by significant immune responses that lead to graft rejection.^8^ Synthetic vascular grafts represent another material for bypass and demonstrate long-term patency for large- and medium-diameter vessels but have not yet demonstrated clinical efficacy in arteries with a diameter smaller than 6 mm.^9^^,^^10^ Expanded polytetrafluoroethylene (ePTFE) is used globally in large-diameter vascular grafts. However, the hydrophobic surface of ePTFE reduces endothelial cell adhesion, leading to platelet activation, thrombosis, and decreased compliance in small-diameter grafts.^11^ To circumvent these issues, various surface augmentations of synthetic grafts have been attempted. These include both passive and active modifications.^8^ Passive modifications attempt to improve graft endothelialization and consist of techniques like incorporating vascular growth factors and antibodies.^8^ Active modifications strive to prevent thrombosis. Such methods include polymers that are activated by thrombus and release the urokinase plasminogen activator to dissolve the clot.^12^ While surface modifications are continuing to advance, the problems of thrombosis, intimal hyperplasia, and long-term patency have not yet been optimized.^8^ Other prior studies demonstrate a tissue-engineered 6-mm vascular graft by lining ePTFE synthetic grafts with autologous venous endothelial cells.^13^^,^^14^ However, the generation of patient-specific tissue-engineered grafts is costly and time consuming. In addition, although these engineered grafts were able to maintain 60% primary patency for more than 10 years in a clinical study,^14^ the patency rate is 20% lower than that of arterial grafts.^15^^,^^16^

Pluripotent stem cells (PSCs) present a promising path to address this challenge thanks to two intrinsic characteristics: the ability to self-renew by cell division and the capacity to differentiate into any cell type that constitutes the human body.^17^^,^^18^^,^^19^^,^^20^ This combination makes PSCs an invaluable and scalable resource for vascular tissue engineering by providing an unlimited supply of homogenous cells. Previously, we have successfully generated arterial endothelial cells (AECs) from PSCs.^21^ Compared to venous endothelial cells, these AECs demonstrated higher nitric oxide production and lower leukocyte adhesion, which are critical to maintain the patency of arteries. Moreover, several studies have demonstrated that inactivating major histocompatibility complex (MHC) class I and II genes promotes engraftment of allogeneic PSC-derived cells.^22^^,^^23^^,^^24^^,^^25^ This finding opened an opportunity to generate hypoimmunogenic AECs from PSCs for tissue engineering “off-the-shelf” small-diameter arterial grafts with improved long-term patency.

Although small animal models have been widely used for examining the patency of vascular grafts,^26^^,^^27^ they have inherent limitations. For example, while even acellular vascular grafts with 1 mm diameter were able to maintain 73%–95% patency for 6 months in small animal models, acellular vascular grafts with a diameter of 5 mm or less failed in clinical applications.^10^^,^^28^ Non-human primates provide a superior model for investigating vascular grafts because of their genetic, immunologic, metabolic, and physiologic similarities with humans and the ability to use equipment and techniques developed for human applications for vascular implantation and monitoring.^29^^,^^30^

In this study, we established a lower extremity arterial interposition grafting model in rhesus macaques. We then generated 3-mm-diameter artery grafts by lining Food and Drug Administration (FDA)-approved ePTFE with PSC-derived AECs and evaluated their function in this model. We assessed the patency of three types of grafts: naked ePTFE, MHC mismatched unmodified-wild type (MHC-WT) AEC-ePTFE, and MHC class I/II double knockout (MHC-DKO) isogenic AEC-ePTFE. Together, the results demonstrate the successful generation of PSC-derived MHC-WT 3-mm artery grafts for allogeneic arterial revascularization.

## Results

### Generation of high-purity AECs

**Figure 1:**
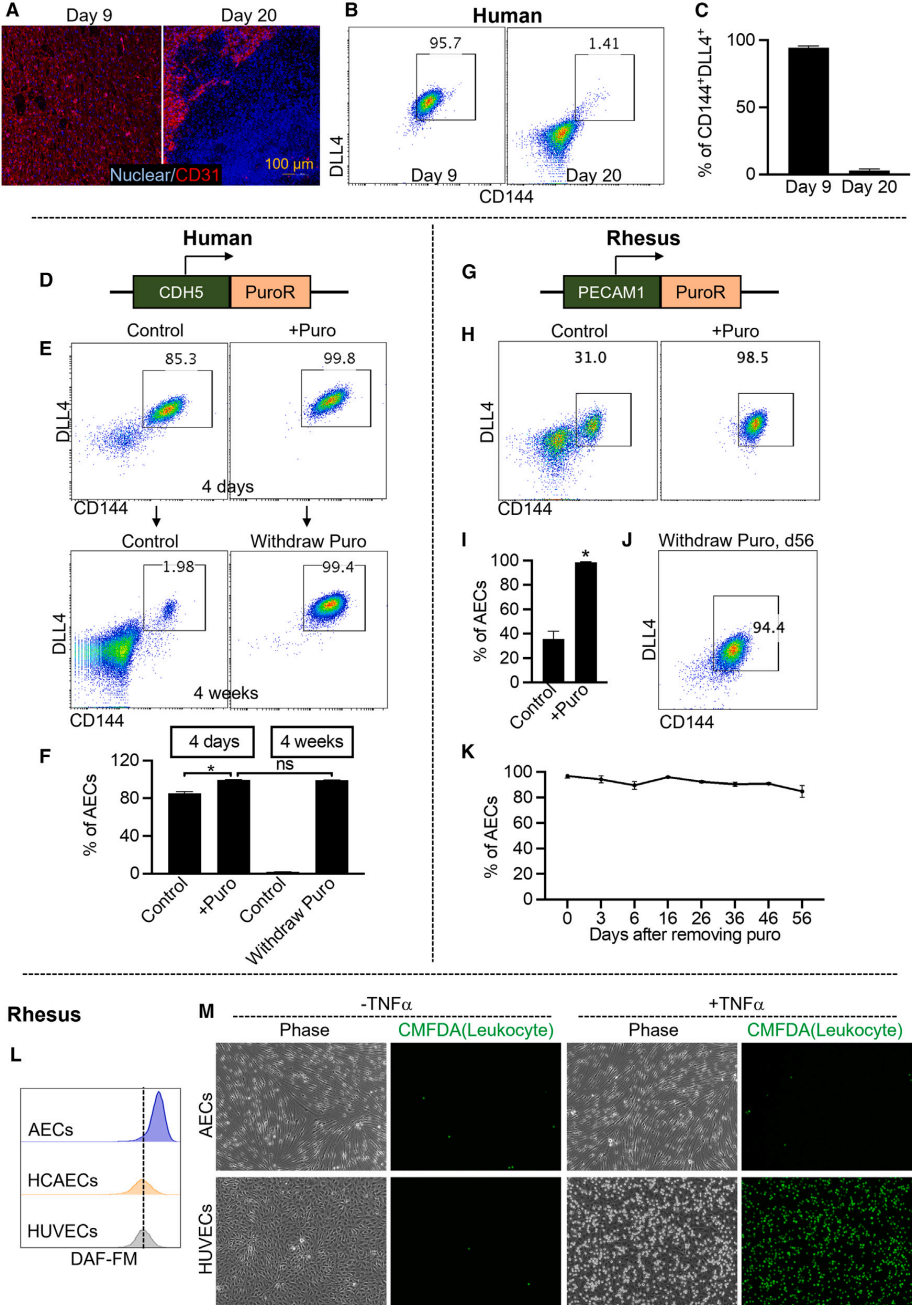
Generation of high-purity AECs by engineering puromycin-resistant cell lines (A) PECAM1 immunostaining of AECs cultured for 9 days and 20 days without passaging. (B) Representative flow cytometry dot plots showed expression of CD144 and DLL4 in day 9 and day 20 cultures. (C) Percentages of CD144^+^DLL4^+^ AECs at day 0 and day 20. Data are represented as mean ± SD. *n* = 3 biological replicates. (D) Schematic of human *CDH5*-PuroR cell line. (E) Upper, representative flow cytometry dot plots show expression of CD144 and DLL4 with or without puromycin treatment for 4 days. Lower, representative flow cytometry dot plots show expression of CD144 and DLL4 after withdrawing puromycin for 4 weeks (F) Percentages of CD144^+^DLL4^+^ AECs. Data are represented as mean ± SD. *n* = 4 biological replicates. Student’s t test; ∗, *p* < 0.05; ns, not significant. (G) Schematic of rhesus *PECAM1*-PuroR cell line. (H) Representative flow cytometry dot plots show expression of CD144 and DLL4 with or without puromycin treatment for 3 days. (I) Percentages of CD144^+^DLL4^+^ AECs. Data are represented as mean ± SD. *n* = 4 biological replicates. Student’s t test; ∗, *p* < 0.05; ns, not significant. (J) Representative flow cytometry dot plots show expression of CD144 and DLL4 with or without puromycin treatment for 56 days. (K) Purity of rhesus CD144^+^DLL4^+^ AECs after withdrawing puromycin. Data are represented as mean ± SD. *n* = 3 biological replicates. (L) Nitric oxide production measured by 4-amino-5-methylamino-2',7'-difluorofluorescein diacetate (DAF-FM) staining. HUVECs (human umbilical vein endothelial cells) and HCAECs (human coronary arterial endothelial cells) were used as controls. (M) Leukocyte (labeled by chloromethyl derivatives of fluorescein diacetate [CMFDA]) adhesion assay. Tumor necrosis factor alpha (TNF-α) treatment increased leukocyte adhesion of HUVECs but not AECs. See also Figures S1–S4.

Maintaining long-term purity of the AECs is critical for the generation of artery grafts. Therefore, we assessed long-term cultures of human AECs generated from human PSCs.^17^^,^^21^^,^^31^ We found that the initial AEC purity of 96% was reduced to less than 2% after 20 days of culture without splitting, as evidenced by the expression of the endothelial markers CD31/PECAM1 and CD144/CHD5 and the arterial-specific cell marker DLL4 (Figures 1A–1C).

To maintain high-purity human AECs, we conducted a high-throughput screen to identify small molecules that could specifically inhibit non-AEC growth (Figure S1A). Out of the 20,000 compounds screened, three small molecules, C21, C23, and C33, significantly increased the purity of CD144^+^DLL4^+^ human AECs after four days of treatment (Figures S1B and S1C; Table S1). However, human AEC purity (CD144^+^DLL4^+^) was lost during long-term culture upon withdrawal of small molecules (Figures S1B and S1C). Similar findings were observed in a screen of AECs generated from human endothelial progenitors derived from cord blood.^32^ The small molecule 8510 identified in this screen was found to increase rhesus AEC purity, while purity was rapidly reduced upon withdrawal of it in extended culture (Figure S1D and Table S1).

Ultimately, we took advantage of gene editing to generate high-purity AECs. Using CRISPR-Cas9 technology, the puromycin resistance (PuroR) gene was knocked into the *CDH5* locus of human PSCs, such that puromycin resistance then depends on CDH5 expression in derived endothelial cells (Figures 1D and S2). The treatment of *CDH5-PuroR* AECs with puromycin improved their purity by eliminating cells not expressing CDH5 (non-AECs) (Figures 1E and 1F). More importantly, even after discontinuing puromycin treatment, human AECs maintained a purity of 99% CD144^+^DLL4^+^ for the duration of at least 4 weeks (Figures 1E and 1F). A similar strategy was used to increase purity of AECs derived from rhesus PSCs by knocking the PuroR gene in the *PECAM1* locus such that puromycin resistance is dependent on PECAM1 expression in endothelial cells. *PECAM1-PuroR* rhesus PSCs were then further differentiated into rhesus AECs^21^ (Figures 1G, 1H, and S3). Like human AECs, rhesus AECs also maintained high purity in long-term culture following puromycin treatment (Figures 1J and 1K) and demonstrated arterial-specific functions, including higher nitric oxide production and lower leukocyte adhesion (Figures 1L, 1M, and S4).

### Modification of ePTFE surface improves AEC adhesion

**Figure 2:**
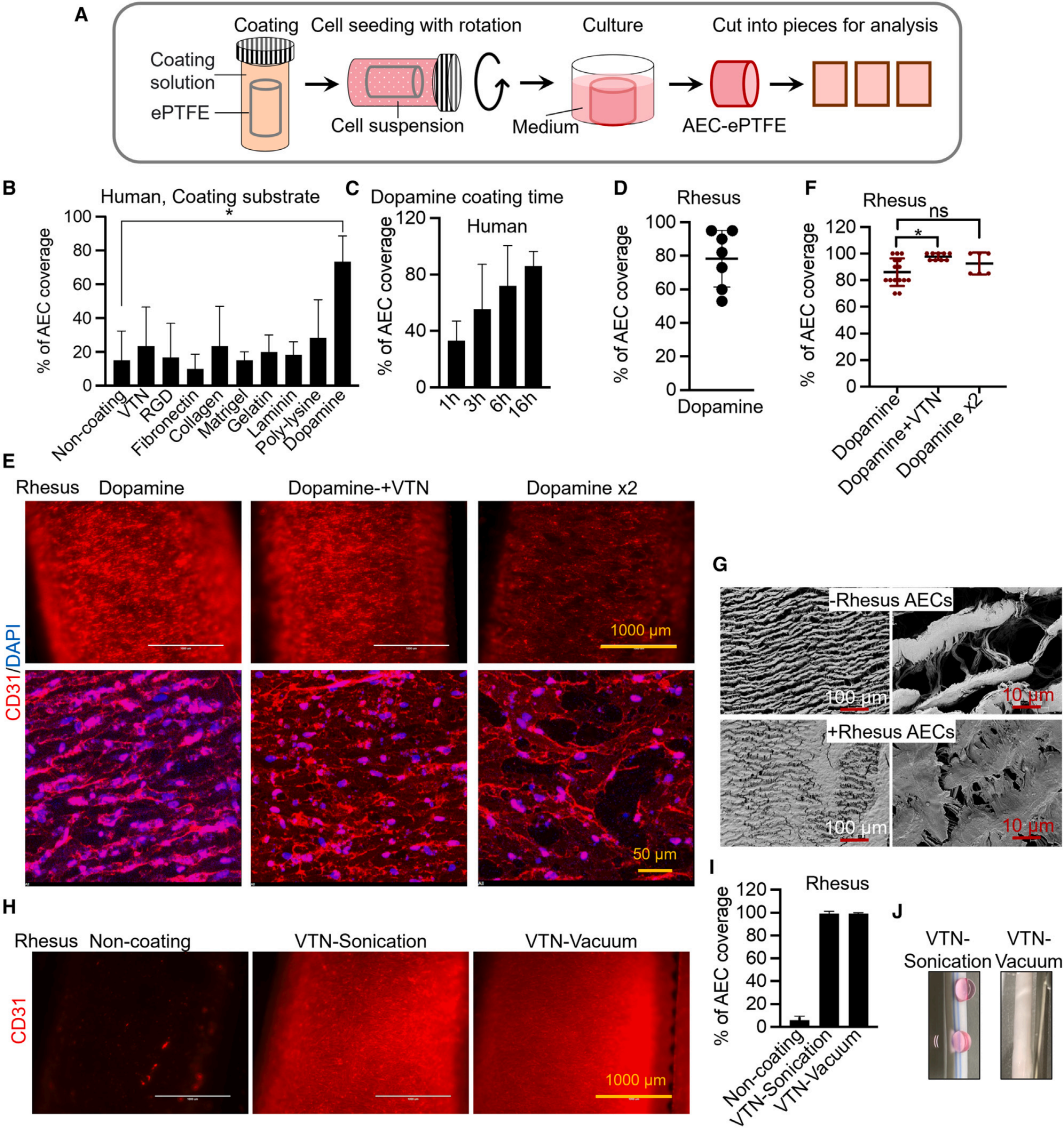
Modifying ePTFE by dopamine and vitronectin or vitronectin-vacuum improves AEC adhesion (A) Schematic of coating, cell seeding, and analysis of AEC-ePTFE. (B) Percentage of human AEC coverage on vitronectin (VTN), RGD peptide, fibronectin, collagen, and dopamine-coated ePTFE. The percentage of cell coverage was calculated by dividing the area occupied by cells by the total surface area. Data are represented as mean ± SD. *n* = 3–6 biological replicates. Student’s t test; ∗, *p* < 0.05. (C) Percentage of AEC coverage on dopamine-coated ePTFE. Data are represented as mean ± SD. *n* = 3–6 biological replicates. (D) Percentage of rhesus AEC coverage on 16-h dopamine-coated ePTFE. Data are represented as mean ± SD. *n* = 7 biological replicates. (E) Representative staining of PECAM1. ePTFE was coated with dopamine, dopamine and VTN, or dopamine twice. (F) Percentage of rhesus AEC coverage on ePTFE. Data are represented as mean ± SD. *n* = 3–6 biological replicates. Student’s t test; ∗, *p* < 0.05; ns, not significant. (G) Scanning electron microscope images of naked ePTFE or rhesus AEC-ePTFE. (H) Representative images of CD31 staining. Rhesus AECs were seeded on ePTFE with VTN coating with sonication or vacuum. (I) Percentage of rhesus AEC coverage. Data are represented as mean ± SD. *n* = 3 biological replicates. (J) Image shows the leakage of VTN-sonication-coated ePTFE compared to VTN-vacuum-coated ePTFE.

The hydrophobic nature of ePTFE makes cell attachment challenging. To address this issue, we explored various coatings for ePTFE, including vitronectin (VTN), RGD-peptide, fibronectin, collagen, Matrigel, gelatin, and poly-lysine. (Figures 2A and 2B). Unfortunately, these coatings were not able to improve human AEC adhesion (Figure 2B). Next, we turned to a dopamine self-polymerization method inspired by the composition of adhesive proteins in mussels.^33^ ePTFE was incubated in a dopamine solution for varied times, ultimately optimized at 16 h (Figure 2C). Dopamine coating resulted in a substantial enhancement of human AEC adhesion (Figure 2B). Unfortunately, in contrast to human AECs, we observed considerable variation in cell adhesion across multiple experiments with rhesus AECs (Figure 2D). To improve rhesus AEC cell seeding, we developed a dual-layered coating approach. This involved incubating the initial dopamine-coated ePTFE with an additional layer of either dopamine or VTN. We found that the second coating by VTN, but not dopamine, significantly improved rhesus AECs adhesion (Figures 2E and 2F). Scanning electron microscope analysis further confirmed rhesus AEC attachment to the surface of ePTFE, validating the dual-layered coating with dopamine and VTN (Figure 2G).

Since VTN is the extracellular matrix used for AEC differentiation, we tried to further simplify the coating by using VTN alone. Because incubation of ePTFE with VTN solution did not enhance AEC attachment (Figure 2B), we applied sonication or vacuum to improve VTN coating. The results showed that VTN coating using both sonication and vacuum increased rhesus AEC coverage to nearly 100% (Figures 2H–2J). Unfortunately, sonication led to the leakage of ePTFE grafts (Figure 2J). Therefore, VTN in combination with vacuum coating was used for further studies.

### Engineering 3-mm-diameter artery grafts that maintain endothelium integrity under physiological flow

**Figure 3:**
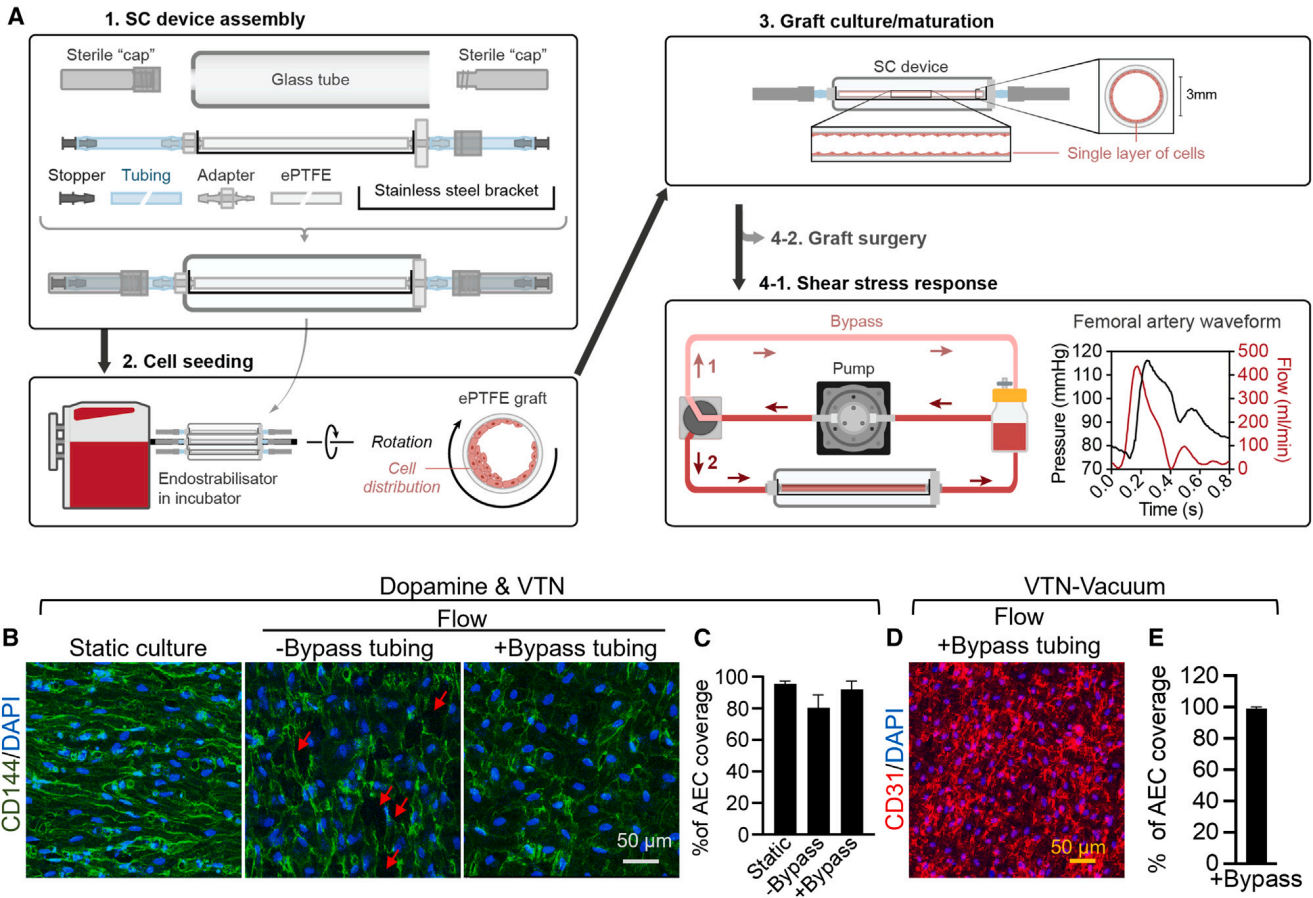
Rhesus AEC-ePTFE artery grafts maintain endothelium integrity under physiological flow (A) Schematic of seeding device assembly, cell seeding, graft culture, and testing of shear stress response. Bypass tubing was used to remove bubbles prior to medium flowing through rhesus AEC-ePTFE grafts (4-1). (B) Representative images of CD144 and DAPI staining in static and flow culture. Static culture: cultured for 8 days in static condition; Flow: cultured for 7 days in static condition and followed by 1 day in flow. Arrows indicate the area that was not covered by rhesus AECs. (C) Percentage of rhesus AEC coverage. Data are represented as mean ± SD. *n* = 6 images from 3 biological replicates. (D) A representative image of CD31 and DAPI staining in flow culture with bypass tubing. (E) Percentage of rhesus AEC coverage. Data are represented as mean ± SD. *n* = 6 images from 3 biological replicates. See also Figure S5.

To facilitate the generation of artery grafts, we designed a customized seeding/culture device (Figure 3A). This device simplified the artery graft production by allowing cell seeding, graft maturation, and testing of the response to shear stress within a single device. Artery grafts were generated by lining ePTFE with AECs. Specifically, the seeding/culture device was assembled as shown in Figure 3A1. The cell suspension was loaded into the inner lumen of the coated ePTFE assembled in seeding/culture device and then placed in a seeding machine with rotation to allow the cells to evenly seed across the inner lumen of the ePTFE (Figure 3A2). After seeding, AEC-ePTFE artery grafts were transferred from the seeding machine to a cell culture incubator for two to eight days in static condition for further maturation (Figure 3A3). To assess the integrity of the endothelium under shear stress, we connected the artery grafts assembled in the seeding/culture device to a bioreactor that was programmed with a physiological femoral artery pressure waveform (Figure 3A4-1). To remove bubbles generated from the initial perfusion, we designed a bypass flow system (Figure 3A4-1). The flow was directed through the bypass flow system and run for 2 to 4 h. Once the bubbles disappeared from the tubing, the flow was redirected to artery grafts. The results show that the bypass system prevented cell detachment due to bubble formation and reduced the experimental variation (Figures 3B and 3C). After one day of exposure to physiological shear stress, rhesus AEC-ePTFE grafts (dual-layer coating of dopamine and VTN) demonstrated the ability to maintain endothelium integrity (Figures 3B and 3C). In addition, VTN-vacuum-coated rhesus AEC-ePTFE grafts were also able to maintain intact endothelium under physiological flow (Figures 3D and 3E).

### Engineering hypoimmunogenic 3-mm artery grafts

Since reduced immune rejection will prolong cell survival, we hypothesized that hypoimmunogenic MHC class I and class II molecule knockout AECs may be beneficial for the artery graft implantation in allogeneic settings. To test this hypothesis, we generated isogenic MHC-DKO AECs from *PECAM1*-puro rhesus PSCs using CRISPR-Cas9 to knockout *B2M* and *CIITA* genes (Figures S5A–S5C). MHC-DKO rhesus AECs demonstrated the lack of MHC class I and II gene expression (Figure S5D). Vascular grafts lined by MHC-DKO rhesus AECs demonstrated similar endothelial lining integrity and cell adhesion strength compared to grafts lined by MHC-WT rhesus AECs with dual-layer coating of dopamine and VTN (Figures S5E and S5F).

### The 3-mm artery grafts lined by MHC-WT AECs display 100% patency in rhesus macaques

**Figure 4:**
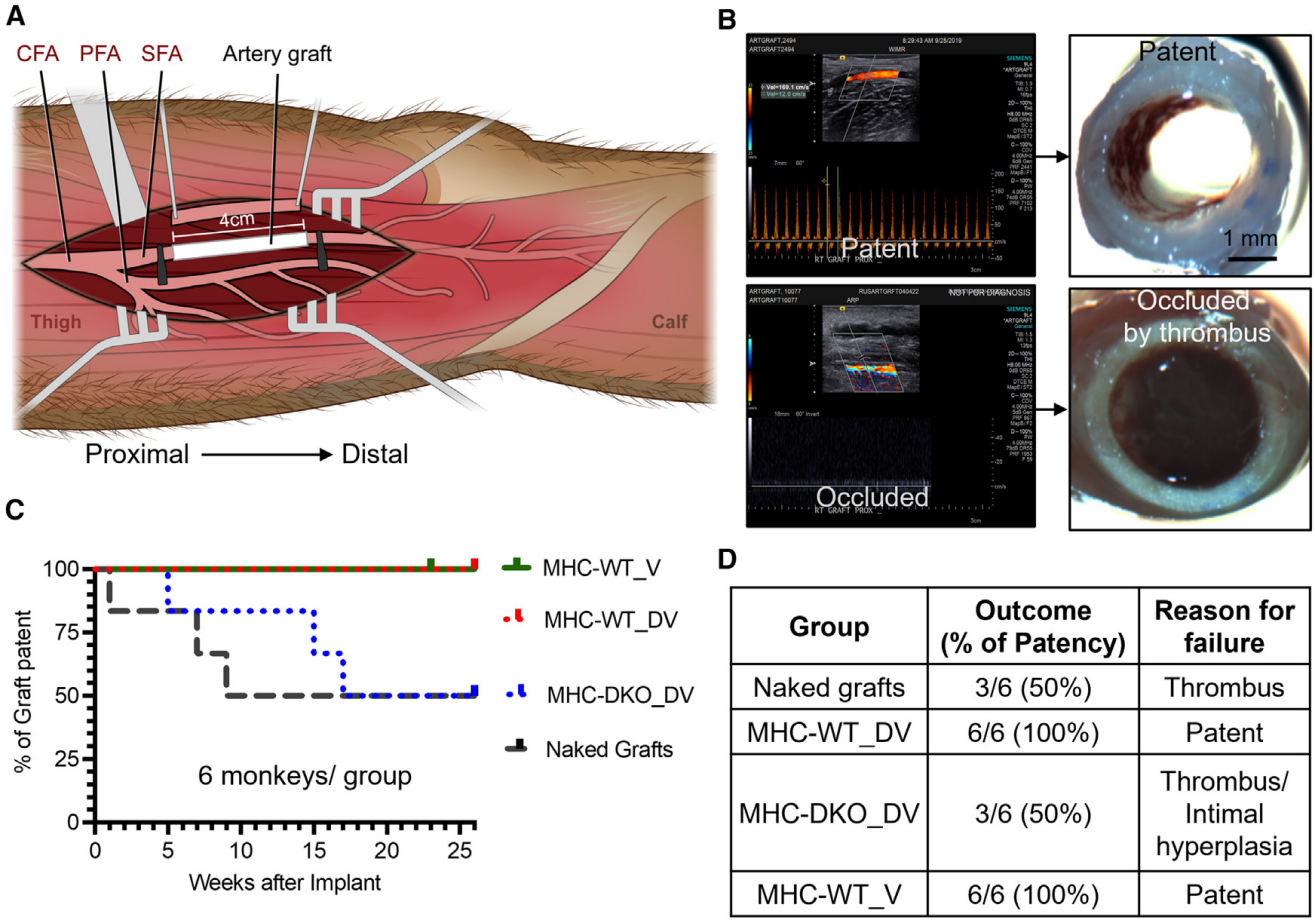
Allogenic MHC-wild-type artery grafts maintain 100% patency in a rhesus model (A) Artery graft bypass surgery model in rhesus monkey. CFA, common femoral artery; PFA, profunda femoral artery; SFA, superficial femoral artery. (B) Representative ultrasound morphology images of occluded and patent grafts. (C) Graft patency as determined by ultrasound. *n* = 6 animals/group. MHC-WT_V: MHC-wild-type grafts, VTN-vacuum coating; MHC-WT_DV: MHC-wild-type grafts, dual-layer coating of dopamine and VTN; MHC-DKO_DV: MHC class I and class II double knockout grafts, dual-layer coating of dopamine and VTN. (D) Summary of the patency and the reason for failure. See also Figures S6 and S7.

To test the patency, durability, and the importance of MHC matching in our artery grafts, we developed an allogeneic rhesus macaque model for lower extremity arterial bypass in the absence of immunosuppression. The 3-mm ePTFE grafts were surgically interpositioned into the superficial femoral artery of rhesus macaques (Figure 4A; Table S2). Patency was visualized by ultrasound bi-weekly and explanted at occlusion (as observed by ultrasound) or at 6-month study endpoints (Figure 4B). Using this model, we assessed graft failure rates in naked grafts (ePTFE without an AEC lining), grafts lined with MHC-WT rhesus AECs, and grafts lined with MHC-DKO rhesus AECs (six animals/group). At the 6-month study endpoint, 50% of naked ePTFE grafts remained patent while the other 50% of grafts failed by occlusion within 9 weeks, primarily through thrombosis (Figures 4B–4D). In contrast, 100% of grafts with a dual-layer of dopamine and VTN coating, lined with MHC-WT rhesus AECs (designated as MHC-WT_DV), remained patent until the 6-month study endpoint (Figures 4B–4D). Unexpectedly, 50% of grafts with a dual-layer of dopamine and VTN coating, lined with DKO-WT rhesus AECs (designated as DKO-WT_DV), occluded by thrombosis or intimal hyperplasia within 17 weeks post-implantation (Figures 4B–4D).

To confirm the capacity of artery grafts lined with MHC-WT rhesus AECs to maintain patency over a prolonged time, an additional cohort of six MHC-WT artery grafts with VTN-vacuum coating (designated as MHC-WT_V) was added to the study. The result demonstrates that MHC-WT_V artery grafts maintained 100% patency for 6 months after implantation (Figure 4C). Overall, these findings suggest that both MHC-WT_DV and MHC-WT_V groups (hereafter referred to simply as MHC-WT artery grafts) could be used for allogeneic arterial revascularization.

Given the failure rate of MHC-DKO AEC grafts, we investigated possible immune interactions. Since MHC class I-negative cells can be lysed by natural killer (NK) cells through “missing self” recognition,^14^^,^^15^^,^^16^^,^^17^ we performed NK killing assays. MHC-DKO AECs were more sensitive to NK cells compared to MHC-WT AECs in both rhesus and humans (Figures S6, S7A, and S7B). To further confirm these results *in vivo*, we transplanted human AECs into NSG-SGM3-hCD34^+^ humanized mouse models. Consistent with *in vitro* results, MHC-WT AECs showed better survival than MHC-DKO AECs in this mouse model (Figure S7C). These results indicate that NK cells could play a major role in mediating the immune rejection to MHC-DKO AECs.

### 3-mm artery grafts undergo moderate and stable remodeling

**Figure 5:**
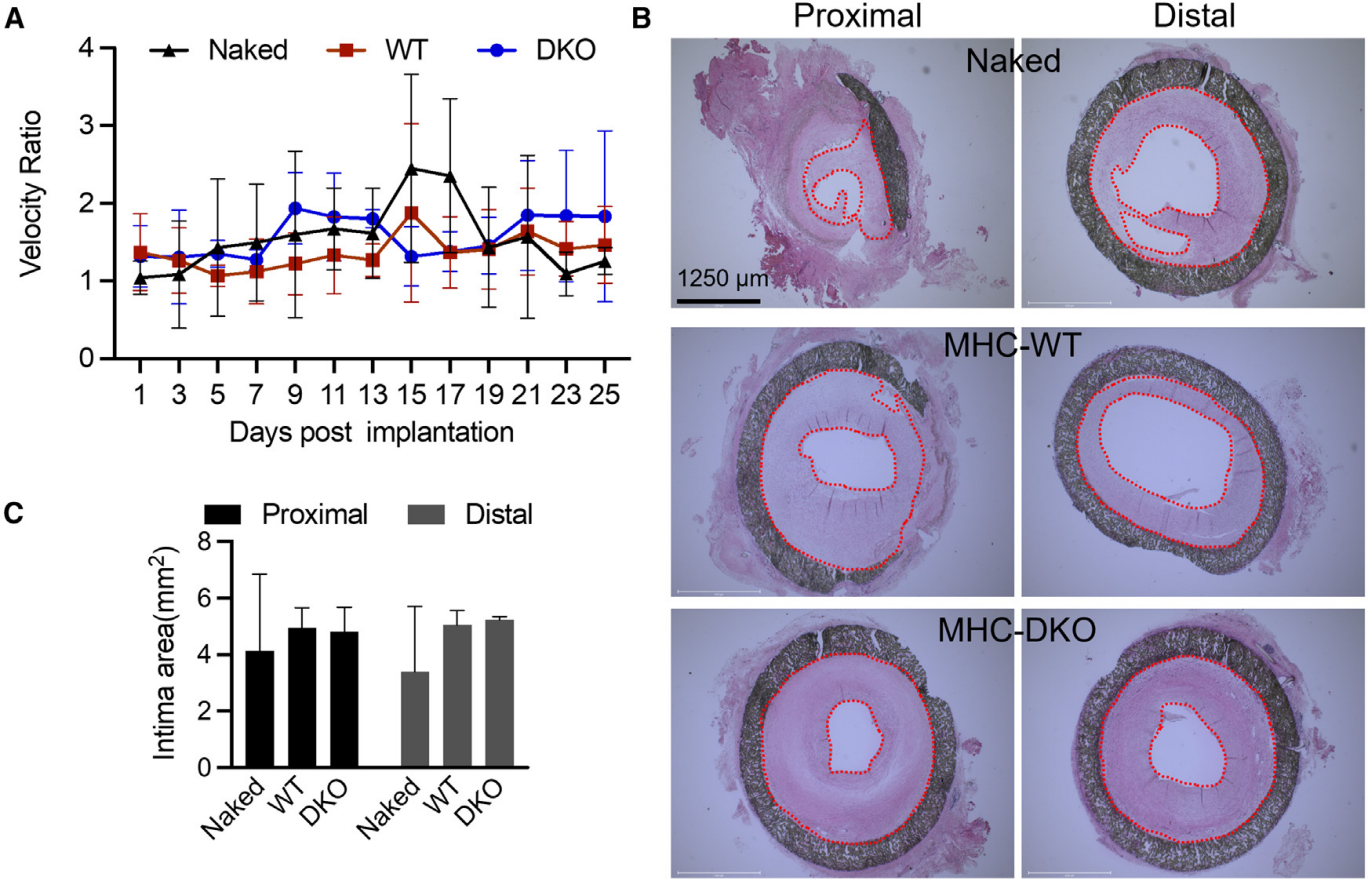
MHC-WT grafts undergo moderate and stable remodeling (A) Blood flow velocity ratio. Due to the constraints of the COVID-19 pandemic, some animals were not subjected to ultrasound monitoring. Therefore, only those grafts that remained patent and were consistently monitored over a period of 6 months have been included in the statistical analysis. The grafts that were not monitored by ultrasound during pandemic are not shown here. Naked, *n* = 3 animals. WT, *n* = 6 animals. DKO, *n* = 3 animals. (B) H&E staining shows the intimal hyperplasia. (C) Statistics data of intima area. Data are represented as mean ± SD. Naked, *n* = 3 animals. WT, *n* = 12 animals. DKO, *n* = 3 animals. See also Figures S8–S10.

Excessive intimal hyperplasia will lead to stenosis of grafts. However, moderate remodeling, including intimal hyperplasia, is necessary for vein grafts to adapt to the arterial environment and thus ensure the long-term patency of vein grafts for artery reconstruction.^1^ To gain insight into the progression of cellular remodeling in artery grafts, we used ultrasound imaging to measure the stenosis ratio (abnormal peak velocity divided by the closest normal velocity) throughout the experiments. A velocity ratio less than two correlates with low risk of graft failure.^34^ The results reveal substantial fluctuations in the velocity ratio for both naked and MHC-DKO grafts over the course of the experiment (Figure 5A). In contrast, the velocity ratio within MHC-WT grafts exhibited sustained stability, consistently staying below 1.5 throughout the experiment, with a temporary increase observed in week 15 (Figure 5A). The velocity ratios of the native vessels prior to implant were below 2 for all groups (Figure S8A). This stable ratio indicates a stenosis level of less than 25%, suggesting moderate remodeling. Given the diameter discrepancy between artery grafts and rhesus femoral arteries (3 mm vs. ∼2.5 mm, respectively [Figure S8B]) and their mechanical mismatch, the occurrence of moderate remodeling is expected and potentially beneficial for adaptation to artery-like conditions. Histologic examination of the grafts that were patent for 6 months confirmed the presence of the intimal hyperplasia, which was mainly located at the proximal and distal sites of the grafts. The extent of intimal hyperplasia was similar across all groups of implants (Figures 5B and 5C). Trichrome staining of graft sections revealed less collagen accumulation and more myofibrils in the MHC-WT group compared to the MHC-DKO group (Figure S9). Infiltration of the MHC-WT or MHC-DKO grafts with leukocytes (CD45^+^ cells) and macrophages (CD68^+^ cells) was not observed within the intima, including a subendothelial location at the study endpoint. These cells were found only within organizing thrombus in animals with graft failure (Figure S10).

### Repopulation of endothelium by host cells in artery grafts

**Figure 6:**
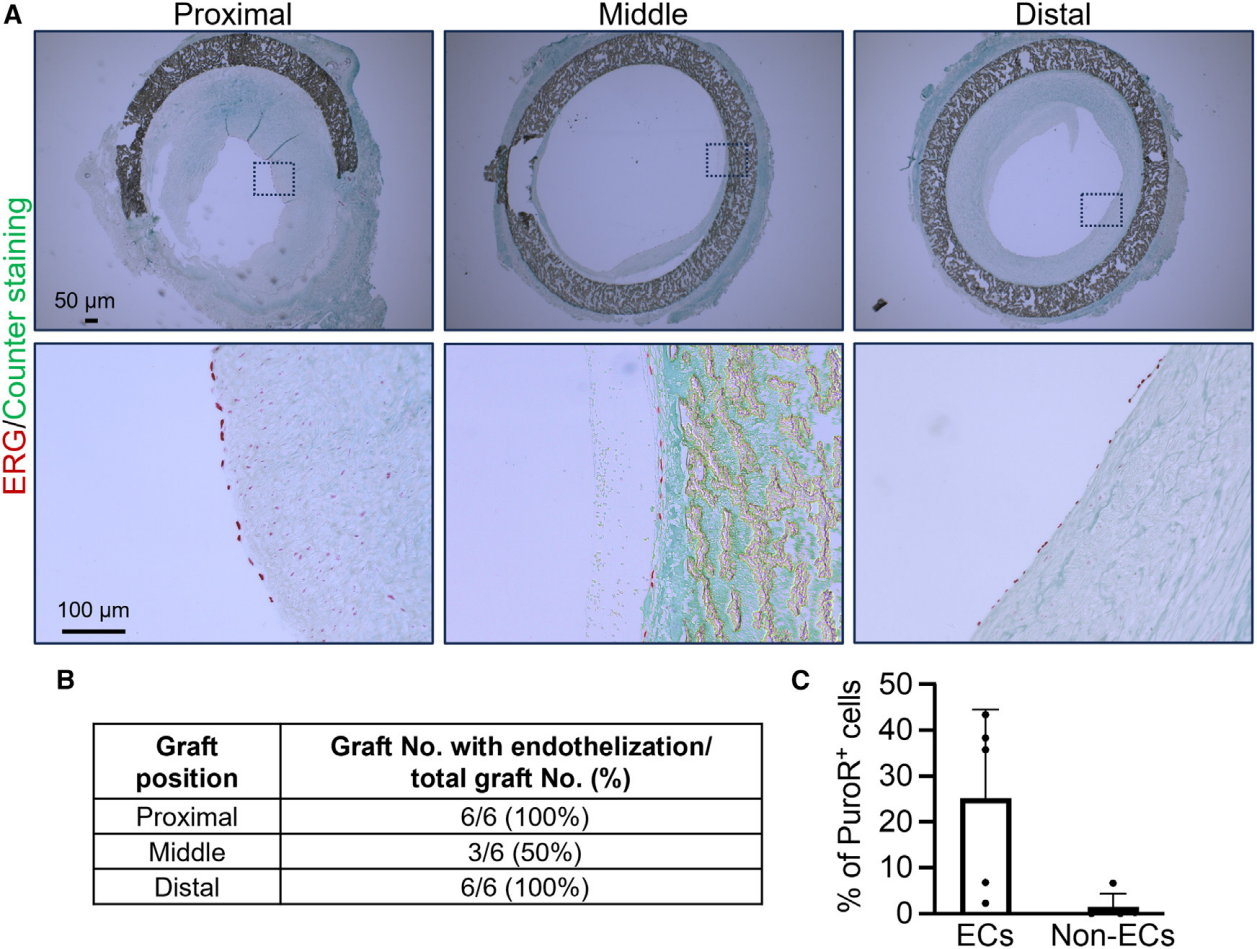
Repopulation of endothelium by host cells in artery grafts (A) Immunostaining of ERG shows the presence of endothelium. (B) Summary of the endothelization in MHC-WT grafts. (C) qPCR analysis of percentage of PuroR^+^ cells that were isolated from MHC-WT grafts. Data are represented as mean ± SD. *n* = 5 animals. See also Figure S11.

Studies in both monkeys and humans have shown that the endothelium of allografts is repopulated with 100% host cells or the mosaic of both donor and host cells.^30^^,^^35^ In our study, immunostaining of ERG (an endothelial cell-specific transcription factor) showed evidence of endothelial cells within MHC-WT artery grafts at 6 months (Figures 6A and 6B), which is similar to native artery (Figure S11). To investigate whether these endothelial cells were repopulated by host (PuroR^−^) cells, we sorted endothelial cells (CD144^+^) and non-endothelial cells (CD144^-^) from the MHC-WT artery grafts and isolated the genomic DNA. qPCR analysis revealed that only a small portion (25%) of endothelial cells was PuroR^+^ donor cells, indicating that most of the endothelial cells were repopulated by the host (PuroR^−^) (Figure 6C). All non-endothelial cells were derived from host cells (Figure 6C), suggesting that donor endothelial cells were resistant to endothelial-to-mesenchymal transition and thus able to maintain their cell fate. In summary, our results suggest that the endothelial repopulation by host cells could contribute to long-term patency of the 3-mm MHC-WT artery grafts.

## Discussion

In this study, we addressed three main challenges for successful generation of small-diameter artery grafts: efficacy, immune rejection, and safety. Prior studies have demonstrated that a functional endothelial layer is critical for vascular grafts to maintain long-term patency by preventing thrombosis and intimal hyperplasia.^36^^,^^37^^,^^38^^,^^39^^,^^40^ However, the development of endothelialized artery grafts for clinical applications poses significant challenges, including uniform endothelial cell seeding, the sourcing of endothelial cells, and generating both universally compatible and arterial-specific endothelial cells.^41^^,^^42^^,^^43^^,^^44^^,^^45^ For instance, one clinical study has developed a 6 mm tissue-engineered graft that demonstrated patency comparable to that of autologous native vein grafts.^13^^,^^14^ But in general, the harvesting of patient’s cells for such engineering is invasive, costly, and time-intensive, with variability across different patients.^13^^,^^14^

To address these challenges, we established technologies for generation of AECs from PSCs and their uniform seeding onto synthetic ePTFE vascular grafts. AECs were capable of long-term maintenance of arterial identity in culture, and AEC-ePTFE artery grafts were able to maintain endothelium integrity under physiological flow. Moreover, we demonstrated the feasibility of using MHC-WT AECs in an allogeneic setting. MHC-WT artery grafts tested in our limb-sparing rhesus macaque arterial transplant model have demonstrated remarkable efficacy, maintaining 100% patency for a minimum of 6 months in non-MHC matched animals. Given the immunological barriers to the clinical usage that restrict the transplantation of allogeneic cells, we also engineered PSCs to mitigate immune response. Deletion of MHC class I and II genes has been demonstrated to reduce the immune response and promote transplanted cell survival.^22^^,^^23^^,^^24^^,^^25^ Surprisingly, in our study, the patency of MHC-DKO grafts was similar to that of naked PTFE graft (50% patency). This could be related to rapid elimination of MHC-knockout cells by natural killer cells.^24^^,^^25^ Additional modifications could be made to the AECs to mitigate NK killing similar to CD47 overexpression or similar strategies.^24^^,^^25^^,^^46^ The higher patency of MHC-WT artery grafts could be due to longer survival of WT AECs because of the extended time required for the activation of T cell alloresponse,^47^ thus providing better support to re-endothelization of artery grafts with the host endothelial cells. Overall, we demonstrated the potential of MHC-WT AECs as a super donor cell for the generation of artery grafts.

Interestingly, the repopulation of endothelium occurred on the proximal and distal parts of all MHC-WT grafts, while only 50% of the medial portions of the MHC-WT grafts were repopulated (Figure 6). The persistence of patency in these partially repopulated grafts raises intriguing questions. One possible explanation could lie in the fact that damaged endothelium is also observed during the injury and repair process in native arteries.^48^ Therefore, it is possible that neighboring endothelial cells would produce nitric oxide and other antithrombotic factors to inhibit the formation of blood clots, maintaining patency.

All implants that maintained patency for 6 months in this study displayed similar degrees of intimal hyperplasia, although we observed less collagen accumulation within intima from MHC-WT grafts. The potential clinical impact of the intimal hyperplasia in this study needs to be studied further, as intimal hyperplasia can be clinically significant and is the leading cause of vein graft failure.^49^ The velocity ratio within the MHC-WT grafts consistently demonstrating values less than 1.5 suggests a moderate amount of remodeling and a stable degree of stenosis less than 25%. This implies that these grafts would likely exhibit long-term patency and the capacity to adapt to arterial conditions in a clinical setting, but future studies would be beneficial to confirm this. In addition, comparing the degree of MHC-WT intimal hyperplasia and its associated clinical impacts, like significant stenosis, to that of other common grafts like the IMA would be valuable.

Beyond further evaluating the effects of intimal hyperplasia, the impact of other patient comorbidities on the efficacy of MHC-WT grafts should also be examined. The animals in this study did not possess conditions such as hyperglycemia, dyslipidemia, or diabetes. However, such disease processes can challenge vascular tissue engineering due to inflammation, impaired remodeling, altered vessel compliance, and oxidation.^50^ Given the overlap of such comorbidities with ischemic cardiomyopathy, it is important to study the utility of MHC-WT grafts in animals with common cardiovascular pathologies.

A primary safety concern with cell-based therapies is the risk of tumorigenicity or other adverse effects arising from the presence of undifferentiated or incorrectly differentiated cells.^51^ Our results show that even an initial contamination of 4% could lead to 98% impurity at a later stage (Figures 1B and 1C). To address these issues, we have used a puromycin resistance gene strategy to produce highly purified AECs whose purity can be maintained for at least 6 months *in vivo* (Figure 6C). This strategy not only enriches AECs but also ensures the elimination of undifferentiated PSCs.

Finally, the use of FDA-approved ePTFE scaffold in our studies presents two valuable advantages. Even in cases of graft failure due to thrombosis or intimal hyperplasia, the ePTFE scaffold remains intact, allowing for subsequent medical interventions to potentially enhance secondary patency rates. Another advantage of ePTFE grafts is that surgeons are accustomed to its use, so the adoption of this technology would necessitate minimal changes to existing standard procedures.

In conclusion, our study provides a strategy to generate 3-mm-diameter allogeneic artery grafts using PSC-derived MHC-WT AECs. The endothelium repopulation of MHC-WT grafts with host cells underscores the potential of temporary grafts and raises significant considerations about the necessity of long-term engraftment and patient-specific therapies. Consequently, our study propels a reconsideration of current stem cell therapy strategies and opens new avenues for future clinical applications.

### Limitations of the study

The study has some limitations. Although there are six monkeys per group, the small sample size remains a constraint due to the significant individual variability in velocity ratios and intimal area of the grafts. Another limitation is the use of MHC-DKO artery grafts. A more advanced hypoimmunogenic model, such as MHC-DKO combined with overexpression of CD47 or Edimer, would provide valuable insights into whether hypoimmunogenic artery grafts resistant to NK killing could improve long-term patency.

## Resource availability

### Lead contact

Further information and requests for resources and reagents should be directed to and will be fulfilled by the lead contact, Dr. John P. Maufort (jpmaufort@wisc.edu).

### Materials availability

All cell lines generated in this study are available from the lead contact with a completed Materials Transfer Agreement.

### Data and code availability

• All data reported in this paper will be shared by the lead contact upon request.
• This paper does not report original code.
• Any additional information required to reanalyze the data reported in this paper is available from the lead contact upon request.

## Acknowledgments

The authors thank Alicia Williams for editorial assistance and Matthew Stefely for help with the scientific illustrations. The authors offer special thanks to Wisconsin National Primate Research Center services including the scientific protocol implementation staff, surgical technicians, and veterinary services; special thanks to the University of Wisconsin radiology and imaging facility; and thanks to the Morgridge Institute for Research. The authors also thank the University of Wisconsin Translational Research Initiatives in Pathology laboratory, supported by the UW Department of Pathology and Laboratory Medicine; 10.13039/100007923UWCCC (P30 CA014520); and the 10.13039/100000179Office of the Director, 10.13039/100000002National Institutes of Health (S10 OD023526), for use of its facilities and services. This work was supported by 10.13039/100000050National Heart, Lung, and Blood Institute grant U01HL134655 and the Office of the Director, National Institutes of Health, under award number P51OD011106.

## Author contributions

J.Z., J.P.M., D.M.T., D.V., and E.S.P. performed experiments. J.Z., J.P.M., and D.M.T. analyzed results and made figures. W.Z., N.J.A., S.L., P.J.N., E.C.S., R.E.G., and S.O.P. performed surgical procedures and assisted with manuscript preparation. Y.X. designed the cell seeding/culture device. M.D.P. performed the high-throughput screening. R.S. performed data analysis. M.S. created scientific illustrations and assisted with manuscript preparation. I.I.S. performed pathology diagnosis. J.Z., J.P.M., M.E.B., N.C.C., L.-S.T., S.O.P., and J.A.T. designed the research. J.Z., J.P.M., I.I.S., and J.A.T. wrote the paper.

## Declaration of interests

J.Z., J.A.T., and J.P.M. are the inventors of a pending patent disclosed to WARF.

## Declaration of generative AI and AI-assisted technologies in the writing process

During the preparation of this work, the authors used ChatGPT to do grammar check. After using this tool, the authors reviewed and edited the content as needed and take full responsibility for the content of the publication.

## STAR★Methods

### Key resources table

**Table:** 

REAGENT or RESOURCE	SOURCE	IDENTIFIER
**Antibodies**		

CD31-Alexa 594 (clone WM59)	Biolegend	Cat# 303126; RRID:AB_2563303
CD144 (clone 55-7H1)	BD Bioscience	Cat# 555661; RRID:AB_396015
CD144-Alexa 647 (clone 55-7H1)	BD Bioscience	Cat# 561567; RRID:AB_10712766
DLL4-APC (clone MHD4-46)	Miltenyi	Cat# 130-096-560; RRID:AB_10827749
ERG (clone EPR3864)	Abcam	Cat# ab92513; RRID:AB_2630401
HLA-A/B/C-Alexa 488 (clone DX17)	BD Bioscience	Cat# 560169; RRID:AB_1645353
HLA-DR/DP/DQAlexa 647 (clone Tü39)	Biolegend	Cat# 361704; RRID:AB_2563168

**Chemicals, peptides, and recombinant proteins**		

Transferrin	Fisher Scientific	Cat# 2914-HT-001G
Insulin	Sigma	Cat# I9287-5ML
FGF2	In house	N/A
TGFβ1	R&D Systems	Cat# 240-B
BMP4	R&D Systems	Cat# 314-BP
Activin A	R&D Systems	Cat# 338-AC
CHIR99021	R&D Systems	Cat# 4423
VEGFA165	R&D Systems	Cat# 293-VE
SB431542	R&D Systems	Cat# 1614
RESV	R&D Systems	Cat# 1418
L690	R&D Systems	Cat# 0681
GlutaMAX	Life Technologies	Cat# 82043745
Chemically Defined Lipids	Thermo Fisher	Cat# 11905031
rhNodal	R&D Systems	Cat# 3218-ND
Glutathione	Sigma	Cat# G4251

**Experimental models: Cell lines**		

Human: H9 embryonic stem cells	In house/WiCell (WA09)	hPSCRegID: WAe009-A
Macaca Mulatta: r420 embryonic stem cells	In house	N/A

**Experimental models: Organisms/strains**		

Macaca Mulatta	Wisconsin National Primate Research Center (WNPRC)	N/A
Mouse: NSG-SGM3-hCD34^+^	Jackson Laboratories	N/A

**Oligonucleotides**		

See Table S4 for gRNA, primers and probes		N/A

**Software and algorithms**		

Prism 10	GraphPad	N/A

**Other**		

Z220377028 (small molecule C21)	EnamineStore	T6275935
Z64462386 (small molecule C23)	EnamineStore	T6051787
1-Octadecyl-2-methylglycero-3 PC (small molecule C33)	ENZO Bioactive lipid	L-108
Z126978510 (small molecule 8510)	EnamineStore	Z126978510

### Experimental model and study participant details

#### Rhesus macaques

Outbred adult male rhesus macaques (*M. mulatta*), 6–13 years old with weights ranging between 12 and 16Kg, were randomly allocated to groups. All rhesus macaques were housed either in same sex pairs or singularly housed if over 15 kg at the Wisconsin National Primate Research Center. All procedures were performed in accordance with the National Institutes for Health Guide for the Care and Use of Laboratory Animals and under the approval of the University of Wisconsin–Madison College of Letters and Sciences, and Vice Chancellor Office for Research and Graduate Education Centers Animal Care and Use Committee. All surgeries were performed in accordance with Institutional Animal Care and Use Committee guidelines under the supervision of trained veterinary staff. Surgeries were performed with animals under general anesthesia with endotracheal tube.

#### Mice

Humanized female NSG-SGM3-hCD34^+^ mice (14 weeks) were purchased from Jackson Laboratories. All the animals were kept in specific pathogen-free conditions and ventilated cages within an ABSL2 facility as same sex groups. Animals were randomly assigned to experimental groups. The experiments were performed under approval from UW-Madison, Institutional Review Board.

#### Cell lines

Human PSCs are cultured in E8 medium (Thermo Fisher customized DF3S base medium (DMEM/F12, 64 ng/ml L-ascorbic acid-2-phosphate magnesium, 14 ng/ml sodium selenium, and 543 μg/ml NaHCO_3_) supplemented with 100 ng/mL FGF2, 1.7 ng/mL TGF-β1, 20 μg/mL insulin, and 10.7 μg/mL Transferrin) on a Matrigel-coated plate (9 μg/cm^2^, or 500 μg/dish) (BD Biosciences, Cat # 354230, Batch 2104930). Rhesus PSCs are cultured in KS-B (E12) medium (E8 medium supplemented with 1X GlutaMAX, 1X Chemically Defined Lipids, 50 ng/mL rhNodal, and 1.94 μg/mL Glutathione) with MEF (mouse embryonic fibroblast) feeders. For maintenance of rhesus PSCs, a concentration of 1.5x10^5^ MEFs/ml was used. Rhesus PSCs used for AEC differentiation, are cultured on double concentration MEFs at 3x10^5^ MEFs/ml. Cultures were maintained at 37°C, 5% CO2 in a humidified incubator. The experiments were performed under approval from UW–Madison Institutional Review Board.

### Method details

#### Human arterial endothelial cell (AEC) differentiation

To achieve the best differentiation results, human PSCs were split using EDTA at 1:4 ratio two days before differentiation. The cells reached 80–90% confluency two days later. At the day of differentiation (day 0), human PSCs were dissociated using Accutase (Invitrogen) for 5 min at 37°C. To induce mesoderm differentiation, the cells were plated on a vitronectin (VTN)-coated plate (0.9 μg/cm^2^ or 50 μg/plate) at a density of 6 x 10^6^ cells/10-cm dish (or 1.1 x 10^5^ cells/cm^2^) by using E8BAC medium for 2 days (approximately 44 h). To improve cell survival, 10 μM Y27632 was used at day 0. From day 2 to day 6, “five factor” medium was used. At day 6, human AECs were passaged in FVIR medium and cultured for another 4 days (day 6–10) on a VTN-coated plate. Human AECs can also be cryopreserved in FVIR media supplemented with 10% DMSO. For puromycin-resistant AECs, 2.5ug/ml puromycin was added to the medium from day 4 to day 8 (the last two days of the differentiation and the first two days after differentiation). Refer to Table S3 for more information about the medium components.

#### Rhesus AEC differentiation

Rhesus PSCs were dissociated using Accutase for 10 min at 37°C. To induce mesoderm differentiation, the cells were plated on a VTN-coated plate at a density of 1 x 10^6^ cells/10-cm dish (or 1.7 x 10^4^ cells/cm^2^) in E8BAC2 medium for two days (approximately 44 h). To improve cell survival, 10 μM Y27632 was used at day 0. From day 2 to day 6, “five factor” medium was used. At day 6, rhesus AECs were passaged in FVIR medium and cultured for another four days (day 6–10) on a VTN-coated plate. Rhesus AECs can also be cryopreserved in FBS supplemented with 5% DMSO. For puromycin-resistant rhesus AECs, 1 μg/mL puromycin was added to the medium from day 6 to day 9 (the first three days after differentiation). Refer to Table S3 for the medium components.

#### Gene editing of human and rhesus cells

##### Generation of human CDH5-PuroR cell line

The 5′- and 3′-homology arms of CDH5 targeting vector were synthesized by IDT (gBlock) and cloned into a PuroR containing vector. PuroR were inserted into the last exon *CDH5* gene. *CDH5* gRNA was synthesize according to the manufacturer’s instructions (GeneArt Precision gRNA Synthesis Kit, Thermo Fisher). To achieve the best electroporation efficiency, human PSCs were passaged with EDTA (1:4 split) and cultured to reach 80–90% confluency two days before the experiment. On the day of the experiment, human PSCs were resuspended at a density of 1.25 x 10^7^ cells/mL in E8 medium supplemented with 10 μM Y27632. To prepare ribonucleoprotein, 0.6 μg gRNA was mixed with 2 μg Cas9 protein (TrueCut Cas9 Protein v2, Thermo Fisher) in 5 μL E8 medium supplemented with 10 μM Y27632. The mixture was incubated for 10 min at room temperature. Next, a 5.5 μL cell suspension and 4 μg *CDH5*-PuroR plasmid was added to ribonucleoprotein. The electroporation was performed in the Neon Electroporation System (Thermo Fisher) with program 13. Cells were combined from 2 to 4 electroporations and seeded into one 10-cm dish coated with Matrigel. Geneticin (100 μg/mL) was added to the media when cells reached 20% confluency for 5–7 days. Surviving colonies were picked after drug selection and expanded in E8 medium. See Table S4 for more information about gRNAs, primers, and probes.

##### Generation of human B2M and CIITA double knockout cell line

B2M and CIITA gRNA were synthesize according to the manufacturer’s instructions (GeneArt Precision gRNA Synthesis Kit, Thermo Fisher). Human PSCs were resuspended at a density of 1.25 x 10^7^ cells/mL in E8 medium supplemented with 10 μM Y27632. To prepare ribonucleoprotein, 0.3 μg of each gRNA was mixed with 2 μg Cas9 protein (TrueCut Cas9 Protein v2, Thermo Fisher) in 5 μL E8 medium supplemented with 10 μM Y27632. The mixture was incubated for 10 min at room temperature. Next, 5.5 μL cell suspension was added to ribonucleoprotein, and electroporation was performed in the Neon Electroporation System (Thermo Fisher) with program 16. To get single-cell clones without drug selection, 2.5% of the electroporated cells were seeded into one 10-cm dish coated with Matrigel. Single cell-derived clones were picked 1–2 weeks later without drug selection. See Table S4 for more information about gRNAs, primers, and probes.

##### Generation of rhesus PECAM1-PuroR cell line and B2M and CIITA double knockout cell line

The generation of rhesus *PECAM1*-PuroR and B2M and CIITA double knockout was similar to human cells, but the cells were seeded on MEF plates with MEF feeders after electroporation. See Table S4 for more information about gRNAs, primers, and probes.

##### Generation of AEC-ePTFE artery graft with dopamine and VTN coated ePTFE

The ePTFE was immersed in the freshly made dopamine solution (2 mg/mL in 10 mM Tris-HCl, pH = 8.5) in a 15 mL tube for overnight (16 h). The next day, the dopamine-coated ePTFE was gently rinsed with Milli-Q water 5 times. The ePTFE was assembled in the cell seeding/culture device and autoclave for 20 min at 120°C. Next, the pre-warmed sterile VTN solution (5 μg/mL in PBS) was loaded into the autoclaved dopamine-coated ePTFE and incubated at 37°C for 1–3 days. It’s important to ensure that no bubbles are present within the lumen of the ePTFE during dopamine and VTN coating. A cell suspension of 1.5–2 x10^6^ AECs/ml was prepared in pre-warmed FVIR medium. The cell suspension was loaded in the inner lumen of the dopamine and VTN coated ePTFE that was pre-assembled in seeding/culture device. Seeding/culture device was placed in a microprocessor controlled seeding machine (Endostrabilisator, Biegler Electronics, Vienna, Austria), rotating at 4 revolutions per hour (rph) at 37° for 3 h, inside a cell culture incubator. After seeding, grafts were cultured in FVIR medium for 8 days. The medium was changed every two days. The day prior to the implantation experiment, a 0.5 cm section of AEC-ePTFE graft was harvested to access the distribution of endothelial cells. The percentage of cell coverage was calculated by dividing the area occupied by cells by the total surface area.

##### Shear stress testing for AEC-ePTFE artery grafts with dopamine/VTN double coating

After an 8-day culture, the seeding/culture device with AEC-ePTFE was connected to a gear pump system. The gear pump was programmed with a femoral artery waveform to simulate physiological conditions. To reduce the bubbles generated during the initial hours of perfusion, a bypass flow system was designed. The flow was directed through the bypass flow system for 2–4 h. Once the bubbles disappeared from the tubing, the flow was redirected to the graft. One day later, AEC-ePTFE artery grafts were harvested for fixation and staining. The percentage of cell coverage was calculated by dividing the area occupied by cells by the total surface area.

##### Generation of AEC-ePTFE artery graft with VTN-vacuum coated ePTFE

The ePTFE was immersed in pre-warmed sterile VTN solution (5 μg/mL in PBS) and placed in a vacuum desiccator. A vacuum pressure of 250 mbar was applied to the desiccator for 1 h, allowing the VTN solution to penetrate and coat the ePTFE surface. After 1 h, the vacuum was turned off and the ePTFE graft in the VTN solution was left within the sealed desiccator overnight. This allows for further adhesion and stabilization of the VTN coating. The next day, the ePTFE was assembled in the seeding/culture device. The pre-warmed sterile VTN solution was loaded inside the lumen of ePTFE, ensuring the lumen is filled. While the inner lumen is being coated with VTN, the outer surface of ePTFE is exposed to sterile atmosphere for aeration, reducing the risk of leaking. The VTN solution was changed 3 days later. Four days later, a cell suspension of 1.5–2 x10^6^ AECs/ml was prepared in pre-warmed FVIR medium with 5 μg/mL VTN. The cell suspension was loaded in the inner lumen of the VTN coated ePTFE pre-assembled in seeding/culture device. Seeding/culture device was placed in a microprocessor controlled seeding machine from Biegler Electronics, rotating at 4 rph at 37° for 3 h, inside a cell culture incubator. After seeding, grafts were cultured in FVIR medium for two days. The day prior to implantation experiment, a 0.5 cm section of AEC-ePTFE graft was harvested to access the distribution of endothelial cells. The percentage of cell coverage was calculated by dividing the area occupied by cells by the total surface area.

##### Shear stress testing for AEC-ePTFE artery grafts with VTN coating

The AEC-ePTFE artery graft with VTN coating was only cultured for two days before shear stress testing. The experiment procedure is the same as AEC-ePTFE artery grafts with dopamine and VTN double coating.

##### Cell adhesion assay

The AEC-ePTFE 3-mm vascular graft was harvested and cut into 0.5 cm sections. The sections were transferred to 2 mL Eppendorf tubes containing FVIR medium and then centrifuged at 2000g for 5 min. Subsequently, the AEC-ePTFE graft was fixed and stained for further analysis. The percentage of cell coverage was calculated by dividing the area occupied by cells by the total surface area.

##### Immunofluorescence staining

Samples were fixed on the day indicated using 4% paraformaldehyde (PFA) for 10 min. Samples were then washed with PBS for 5 min (3 times), permeabilized with 0.2% Triton X-100 in PBS supplemented with 1% BSA for 10 min, and incubated in primary antibody (diluted in 1% BSA in PBS) for overnight at 4°C. On the second day, cells were then washed with PBS for 5 min (3 times) and incubated with fluorescence labeled secondary antibody (Invitrogen) for 2 h at room temperature. Please refer to KRT for more information about the antibodies.

##### Immunohistochemistry staining

Automated immunohistochemistry was performed on the Ventana Discovery Ultra BioMarker Platform (Ventena Medical Systems). Deparaffinization was carried out on the instrument, as was heat-induced epitope retrieval with cell conditioner 1 buffer (Ventana #950-224), an EDTA based buffer pH 8.4, for 64 min at 95°C. The primary antibody was the anti-ERG rabbit monoclonal antibody (Abcam 92513) diluted 1:100 in DaVinci Green antibody diluent (BioCare Medical #PD900H) and incubated for 60 min at 37°C. Slices were rinsed with reaction buffer (Ventana #950-300), incubated with Discovery OmniMap anti-rabbit horseradish peroxidase (Ventana #760–4311) for 16 min at 37°C, and then rinsed with reaction buffer. Discovery Red detection kit (Ventana # 760-228) was used for visualization. Slides were removed from the instrument, counterstained with light green counterstain for 1 min, rinsed with distilled water, dehydrated by oven drying and dipping in xylene.

##### Histopathological analysis

Sections of arterial grafts were embedded in paraffin wax and cut into cross sections of 10 μm in sequential order and mounted on microscopic slides. Sections were stained using Hematoxylin & Eosin (H&E) and Trichrome. All stains were performed by the TRIP pathology lab (University of Wisconsin-Madison School of Medicine and Public Health, Department of Pathology and Laboratory Medicine). Histological analysis of H&E and Trichrome stains was performed by an independent pathologist at the Wisconsin National Primate Research Center. H&E sections were used to examine intima area ImageJ software. Intima area is defined as the intima between the ePTFE graft and lumen area in mm^2^.

##### Nitric oxide production assay

The endothelial cells were seeded on a vitronectin-coated 24-well plate (1x10^5^ cells/well). Two days later, 1 μM DAF-FM (Life Technologies, cat# D-23844) was added to the medium and incubated for 30 min. The cells were harvested for flow cytometric analysis.

##### Leukocyte adhesion

All endothelial cells were cultured on a vitronectin-coated 24-well plate and treated with or without 10 ng/mL TNFα for 4 h. Subsequently, CMFDA (Fisher Scientific, Cat# C2925) pre-labeled U937 cells (2x106 cells) were suspended in 0.5 mL of fresh medium and added to each well. After 20–60 min, the non-attached cells were removed by washing with medium. Imaging was performed immediately afterward.

##### NK killing assay

hAECs were pre-labeled with CMFDA (Fisher Scientific, Cat# C2925) and seeded on VTN coated 96-well plates at 2 x 10^4^ cells/well. Cells were treated with 50 ng/mL IFNγ. One to two days later, 4-6 x 10^4^ primary human NK cells (CD3^−^CD56^+^ cells) were added to the pre-labeled hAEC seeded plate. Two to 4 h later, the dead cells were removed by washing with PBS 3 times. hAEC survival was measured by comparing NK and hAECs co-cultures with hAECs mono-culture samples. rAECs were pre-labeled with CMFDA (Fisher Scientific, Cat# C2925) and seeded on VTN coated 96-well plates at, 0.6–2 x 10^4^ cells/well. Cells were treated with 50 ng/mL IFNγ. Primary rhesus NK cells (CD3^−^CD8^+^CD16^+^ cells) were added at an effector to target ratio 2:1 one day later. Two to 3 h later, rAEC survival was measured as hAECs.

##### Human AEC *in vivo* survival analysis

The experiments were performed under approval from UW-Madison, Institutional Review Board. Humanized NSG-SGM3-hCD34^+^ mice (14 weeks) were purchased from Jackson Laboratories. Animals were randomly assigned to experimental groups. WT or DKO AECs (2 x10^7^ cells/ml) (luciferase^+^) were mixed with of 1 volume of Matrigel and then injected (100 μL/mouse) into the hind flank of the mice. AEC survival was monitored by bioluminescent imaging using IVIS imaging system at the indicated time points.

##### Lower extremity arterial interposition grafting with ePTFE graft (femoral artery)

The rhesus monkey was placed under general anesthesia without complications. Animals receive aspirin for anti-platelet effect up to 7 days before surgery and up to the life of the graft post-operatively. The dose of aspirin is 3–5 mg/kg administered orally once a day. On the day of surgery, the right lower extremity was prepped and draped in a sterile fashion. An 8-cm incision was made in the medial thigh to expose the femoral vasculature. The femoral artery and vein were carefully exposed using sharp and blunt dissection. The femoral artery was freed from surrounding tissue using microsurgical instruments under magnification. A 3-mm ePTFE graft with or without rhesus AECs was placed in the field. Clamps were applied to the femoral artery for proximal and distal control. The artery segment between the clamps was divided and a small section of the vessel was excised. The cut ends of the superficial femoral artery were shaped to match the graft diameter. The graft was sutured to the artery using 8-0 Nylon sutures, both proximally and distally. Excess diameter was trimmed, and the graft was irrigated with heparinized saline. The arterial clamps were removed to confirm blood flow through the graft. Pulsatile bleeding from the proximal anastomosis was addressed with additional sutures. The wound was closed in layers, and a local anesthetic was injected. The incision was dressed with triple antibiotic ointment.

##### Lower extremity arterial interposition graft explantation

The animal was placed under general anesthesia without complication. The right lower extremity was draped in the usual sterile fashion. A 10-cm incision was made in the medial thigh through the previous surgical incision. A combination of blunt and sharp dissection was used to expose the artery and the graft from the surrounding tissue. The proximal and distal arteries were suture ligated using a 4-0 silk tie. The graft and approximately 0.8cm of artery on each end were excised. The proximal end was tagged with a silk tie. Meticulous hemostasis was achieved, and the wound was washed out with sterile saline. Heparinized saline was used to flush out the explanted graft. The wound was closed in three layers. 4-0 vicryl was used to approximate the subcutaneous tissues and the deep dermal layer in a simple interrupted fashion. 4-0 vicryl was used in a running subcuticular fashion to close skin. Diluted liposomal encapsulated bupivicane (diluted 1:1 with sterile saline) was infiltrated into the subcutaneous tissue surrounding the closure.

##### Flow cytometry

Cells (0.1–1 x 10^6^) were resuspended in diluted antibodies and incubated at 4°C for 0.5–1 h. Cells were then washed with 2% FBS-PBS for 1–2 times and cytometric analysis was performed on BD FACSCanto II. FlowJo was used for the data analysis. Please refer to KRT for more information about the antibodies.

##### High-throughput screening for human AECs and non-AECs

The NOS3-NLuc-Tom reporter human PSC cell line was differentiated into AECs as described above. Non-AECs were obtained by culturing AECs for more than 20 days without passage. Two screens were performed in parallel using AECs and non-AECs.

AECs or non-AECs were seeded on VTN coated (50 μg/plate) 384-well plate (1 x 10^6^ cells/plate) with FVIR medium (Table S3). Two hours later, the compounds were added to the media. The cells were fed every two days, and the compounds were added after each feeding. The screen last for 6 days. For AECs, the Luciferase substrate (1000x dilution) (Promega, Cat# N1120) was added to media for 15 min and luminescence was measured. The cell survival was then determined by alamarBlue assay (Thermo Fisher, Cat# DAL1025) according to the manufacturer’s manual. For non-AECs, only alamarBlue was used to detect cell survival. The small molecules with normalized luciferase reads greater than “average +3 x STDEV” were selected for further measuring of AEC/non-AECs by alamarBlue analysis. Our score criteria for primary “hit” molecules are those that have ratio of AEC/non-AECs greater than 5. Enamine 2011 Representative Diversity Library, ENZO Bioactive lipid, ENZO Epigenetics library, ENZO Ion channel ligand library, ENZO Natural Products Library, Prestwick Chemical Library, The Thomson Custom Stem Cell Modulator 1, GlaxoSmithKline Protein Kinase Inhibitors, and Selleck Kinase Inhibitors were used for screening.

##### High-throughput screening of human eAEPs

Two screens were performed in parallel using cell types previously described.^32^ In the first screen, mesenchymal cells (the progeny of EndoMT-permissive eAEPs) were cultured for three days in the presence of different small molecule compounds alongside controls never exposed to the compounds. In the negative screen, AECs (the progeny of EndoMT-resistant eAEPs) were treated with same conditions as the mesenchymal cells. At the end of three days, wells were scored by AlamarBlue (Thermo Fisher), a dye whose absorbance or fluorescence is linearly correlated with the number of live cells in each well. The readouts of the two screens were compared to identify molecules that inhibit the survival and/or proliferation of the non-AECs without inhibiting AECs. Our score criteria for primary “hit” molecules are those that can reduce the number of mesenchymal cells by 80% or greater without reducing the number of AECs by more than 20%. We screened 22,846 small molecule compounds from our libraries including: Enamine 2011 Representative Diversity Library, ENZO Bioactive lipid, ENZO Epigenetics library, ENZO Ion channel ligand library, ENZO Natural Products Library, Prestwick Chemical Library, The Thomson Custom Stem Cell Modulator 1, GlaxoSmithKline Protein Kinase Inhibitors, and Selleck Kinase Inhibitors.

### Quantification and statistical analysis

All described results are representative of at least three independent experiments. The number of samples (n) was described in detail for each figure panel. Statistical analyses were performed using Prism 10 software (GraphPad). Unpaired Student’s t test was used for the comparison between two groups. Log rank test was used for comparison between patency curves. Results are presented as mean ± SEM unless otherwise indicated. *p* < 0.05 was considered statistically significant.

## Supplemental information

Document S1. Figures S1–S11 and Tables S1–S4

Document S2. Article plus supplemental information
