# Supplementary material for: Small-diameter artery grafts engineered from pluripotent stem cells maintain 100% patency in an allogeneic rhesus macaque model
Source: Cell Rep Med. 2025 Mar 10;6(3):102002. doi: 10.1016/j.xcrm.2025.102002 (PMC11970380; doi:10.1016/j.xcrm.2025.102002)
Supplement: Document S1. Figures S1–S11 and Tables S1–S4 [file mmc1.pdf]

Cell Reports Medicine, Volume *6*

Supplemental information

Small-diameter artery grafts engineered from pluripotent stem cells maintain 100% patency in an allogeneic rhesus macaque model

Jue Zhang, Diana Marcela Tabima, David Vereide, Weifeng Zeng, Nicholas J. Albano, Sarah Lyon, Peter J. Nicksic, Ellen C. Shaffrey, Robert E. George, Mitchell D. Probasco, Elizabeth S. Perrin, Yiyang Xu, Matthew E. Brown, Ron Stewart, Naomi C. Chesler, Lih-Sheng Turng, Samuel O. Poore, Igor I. Slukvin, James A. Thomson, and John P. Maufort

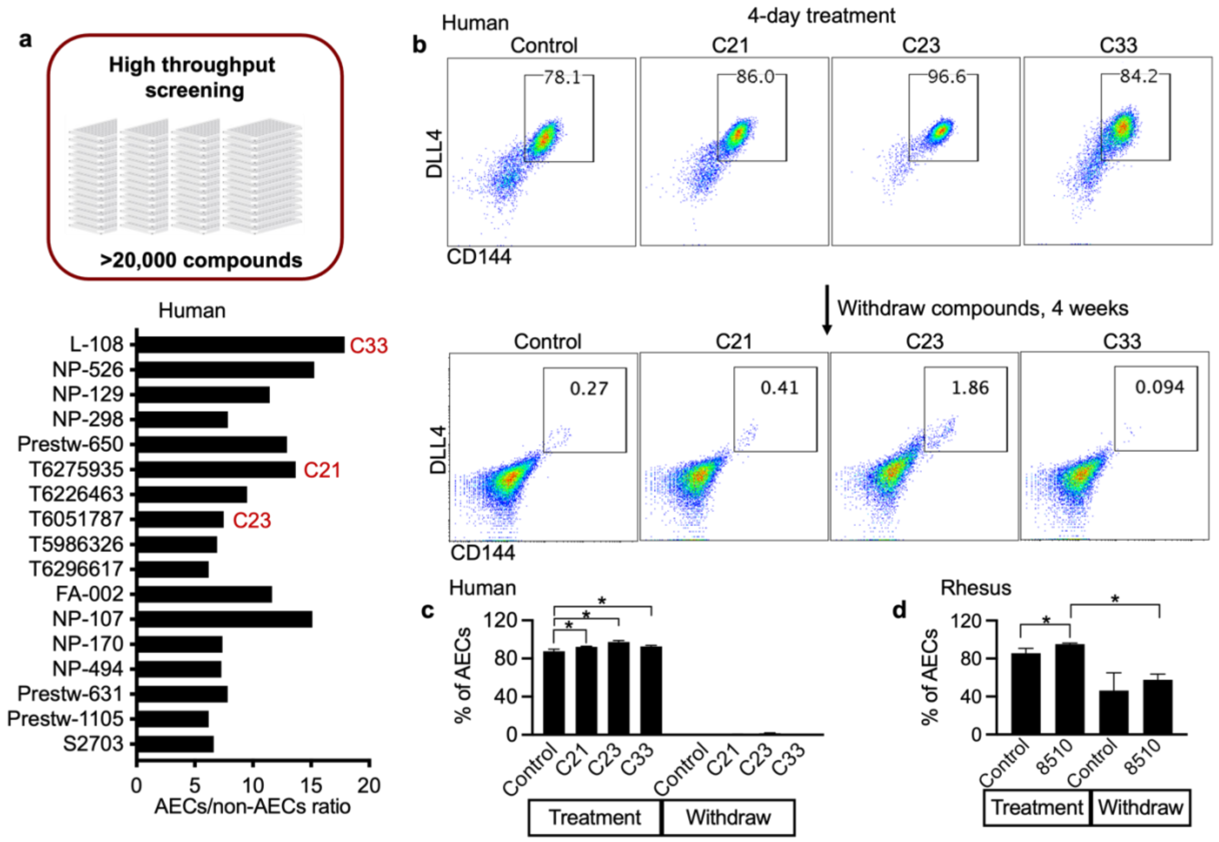

Figure S1: Screening compounds that inhibit non-AECs. Related to Figure 1.

a, High throughput screening. Human AECs and non-AECs ($CD144^{-}DLL4^{-}$ cells) derived from the NOS3-NLuc-Tom cell line were used for the screen. Luciferase was performed and the small molecules with normalized luciferase reads greater than "average + 3 x STDEV" were selected for further measuring of AEC/non-AECs by alamarBlue analysis. The small molecules with AEC/non-AECs ratio > 5 are shown. **b,** Representative flow cytometry expression of CD144 and DLL4 in human AECs before and after withdrawing compounds. **c,** Percentages of $CD144^{+}DLL4^{+}$ human AECs at day 0 and day 20. Data are represented as mean ±SD. *, $p < 0.05$. n=3 biological replicates. **d,** Percentages of $CD144^{+}DLL4^{+}$ rhesus AECs before and after withdrawing compounds. Data are represented as mean ±SD. *, $p < 0.05$. n= 3 biological replicates. More information about the compounds can be found in table 5.

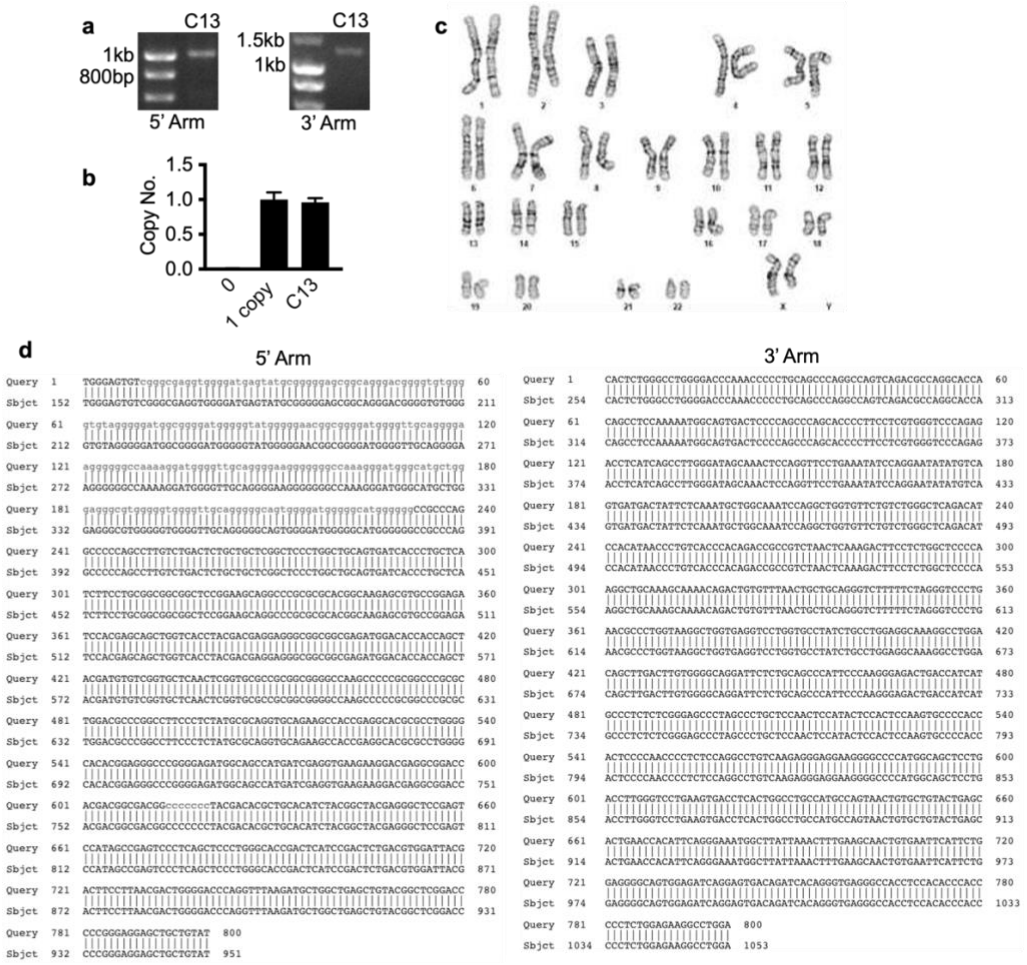

Figure S2. Generation of human *CDH5*-PuroR cell line. Related to Figure 1.
a, Junctional PCR analysis *CDH5*-PuroR knock-in allele. **b**, qPCR analysis of clone 13 (C13) *CDH5*-PuroR copy number. Data are represented as mean ±SD. n = 2 independent experiments. **c**, Karyotyping of *CDH5*-PuroR cells. **d**, DNA-sequencing results of homology arms of *CDH5*-PuroR knock-in allele.

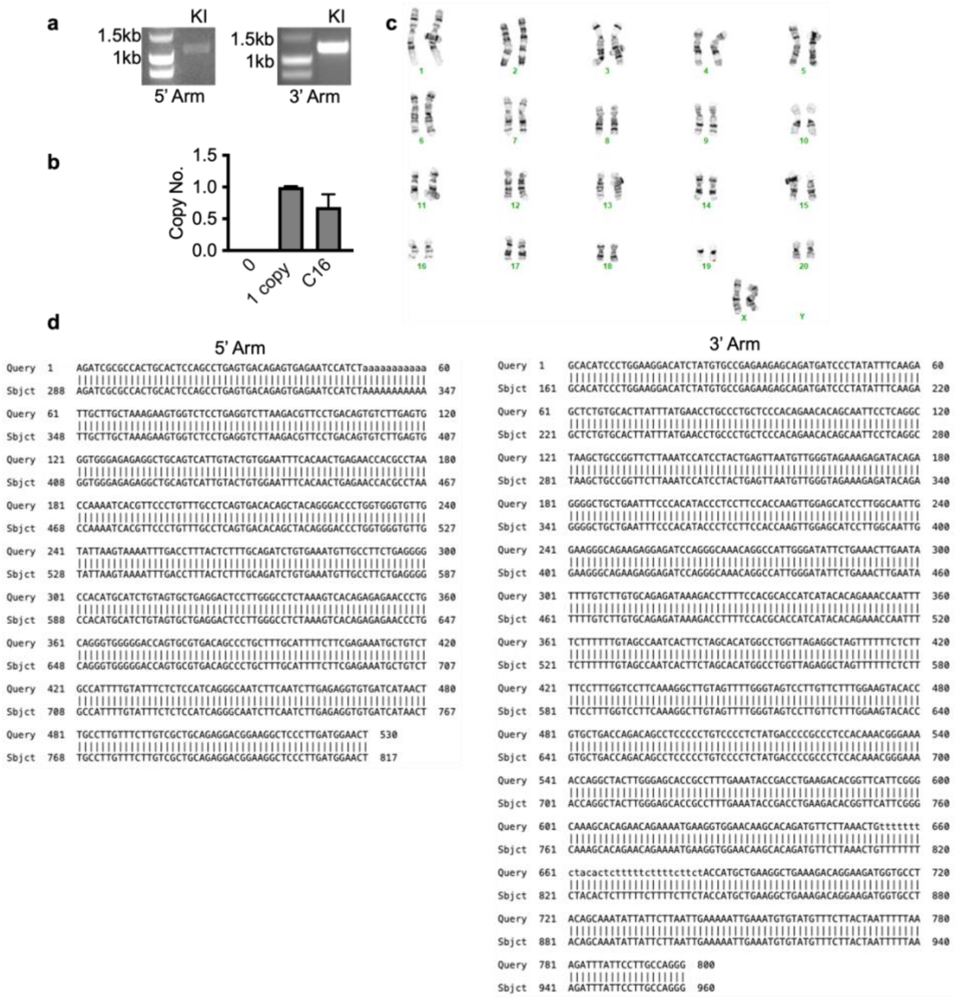

Figure S3. Generation of rhesus *PECAM1*-PuroR cell line. Related to Figure 1.

a, Junctional PCR analysis *PECAM1*-PuroR knock in allele. **b**, qPCR analysis of clone 16 (C16) *PECAM1*-PuroR copy number. Data are represented as mean ±SD. **c**, Karyotyping of *PECAM1*-PuroR cells. **d**, DNA-sequencing results of homology arms of *PECAM1*-PuroR knock-in allele. Note that in panel a, the PCR product of the 5' arm appears larger than the expected size of 988bp. However, the DNA-sequencing results in panel d confirm that the sequence is indeed correct.

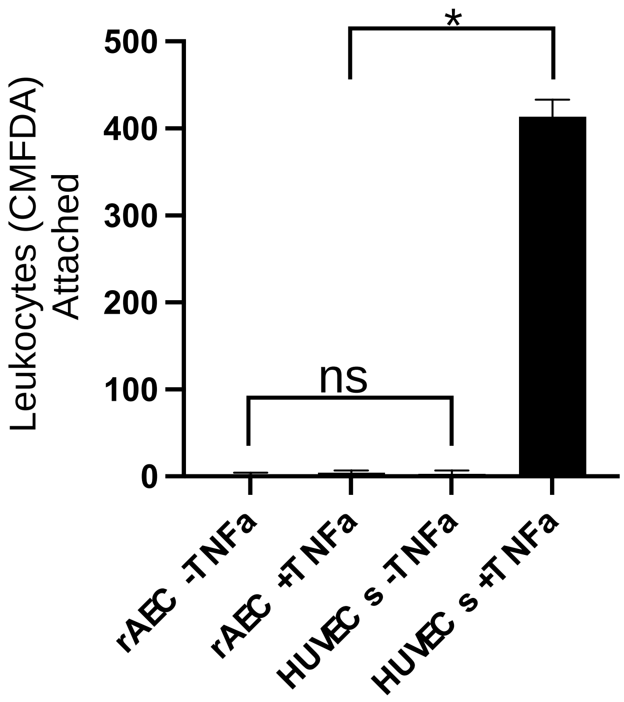

Figure S4. Quantification of leukocyte adhesion assay. Related to Figure 1.
Data are represented as mean ±SD. Student's t-test; *, $p<0.05$. n=6 images from 3 biological replicates.

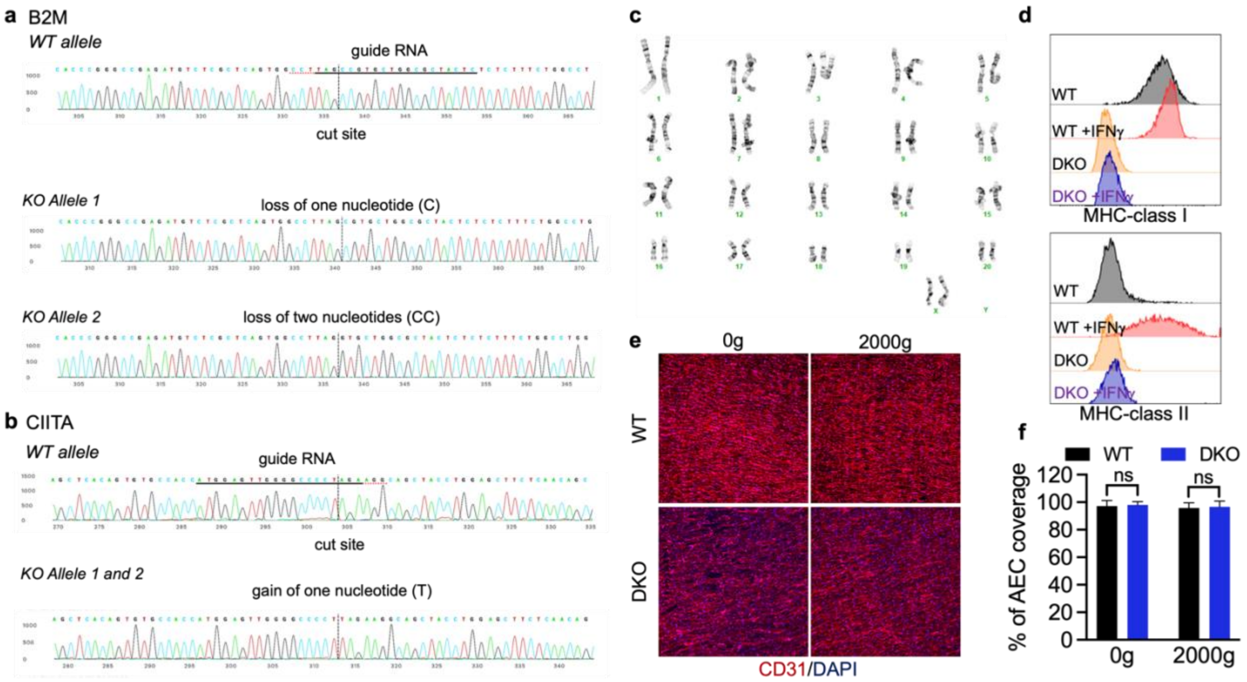

Figure S5. Generation of MHC-DKO cell line from rhesus *PECAM1*-PuroR cell line. Related to Figures 3 and 4.

a, DNA sequencing result shows the deletion of B2M gene. **b**, DNA sequencing result shows the deletion CIITA gene. **c**, Karyotyping of MHC-DKO cells. **d**, Flow cytometric analysis of MHC class I (HLA-A/B/C) and class II (HLA-DQ/DR/DP) gene expression. IFNγ (50ng/ml) was used to treated rhesus AECs for 6 days to enhance MHC gene expression. **e**, Representative images of CD31 and DAPI staining in MHC-DKO AEC-ePTFE graft with or without centrifuge. Centrifuge (gravity = 2000g) was used to test the cell adhesion strength. **f**, Percentage of AEC coverage. Data are represented as mean ±SD. n=6 images from 3 independent experiments.

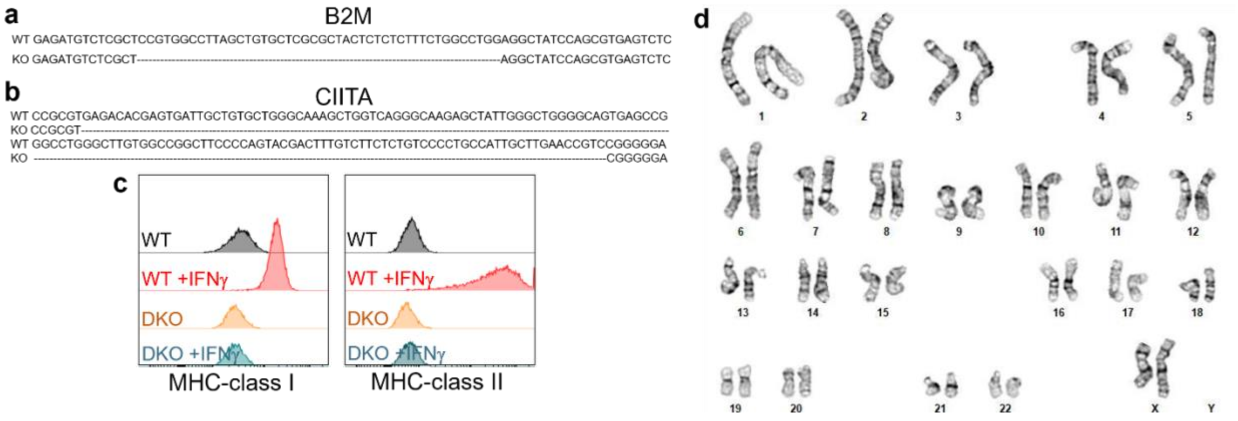

Figure S6. Generation of MHC-DKO cell line from human *CDH5*-PuroR cell line. Related to Figures 3 and 4. **a**, DNA sequencing result shows the deletion of B2M gene. **b**, DNA sequencing result shows the deletion CIITA gene. **c**, Flow cytometric analysis of MHC class I (HLA-A/B/C) and class II (HLA-DQ/DR/DP) gene expression. **d**,) Karyotyping of MHC-DKO cells.

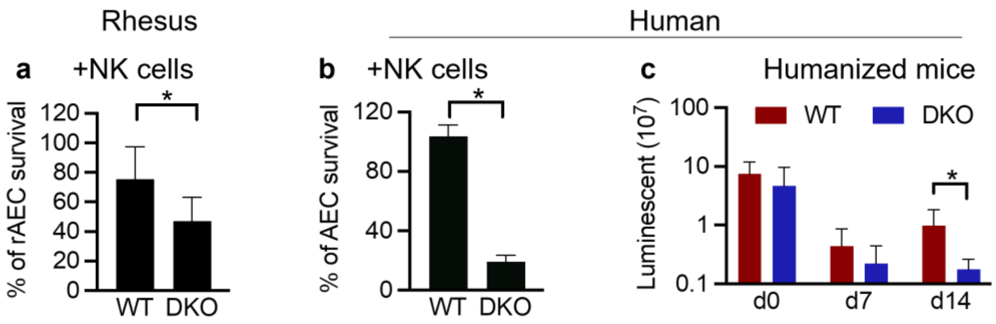

Figure S7. MHC-DKO AECs are more sensitive to NK cells. Related to Figure 4.

a, NK killing assay of rhesus AECS. **b**, NK killing assay of human AECs. **c**. Grafts of luciferase^{+} WT or DKO hAECs were transplanted into allogeneic humanized mice (NSG-SGM3-CD34^{+} mice). Statistics data of hAEC survival was shown by luminescent signals. Data are represented as mean ±SD. Student's t-test; *, p<0.05. n = 5 biological replicates.

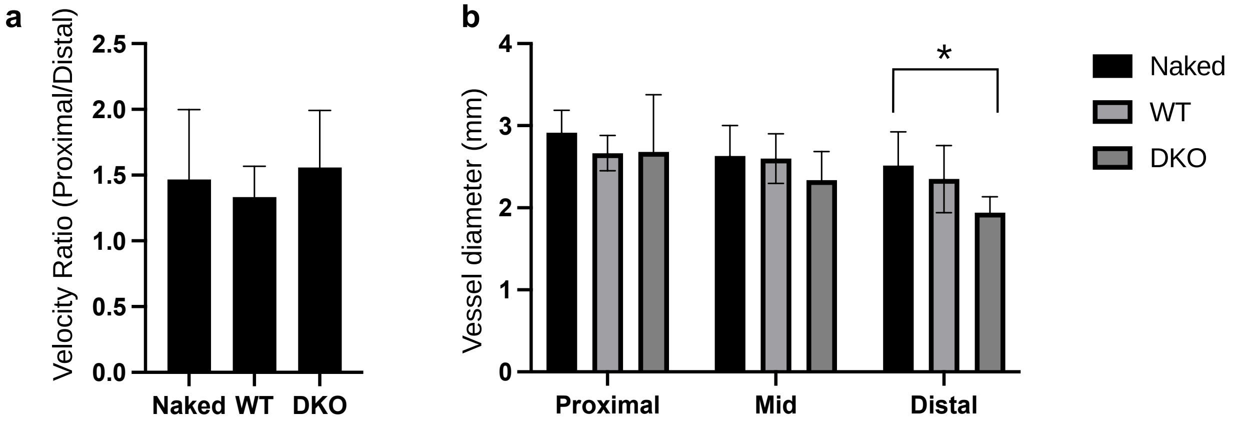

Figure S8. Native artery velocity ratio and vessel diameter. Related to Figure 5.
a, velocity ratio from calculated native artery peak velocities (cm/s) proximal artery/distal artery. Data are represented as mean ±SD. Student's t-test; n.s. n = 6, per group. b, vessel diameters as measured by ultrasound imaging at the proximal, mid and distal graft sites. Only statistical difference is between naked and MHC-DKO grafts at the distal site. Data are represented as mean ±SD. Student's t-test; *, $p<0.05$. n = 6, per group.

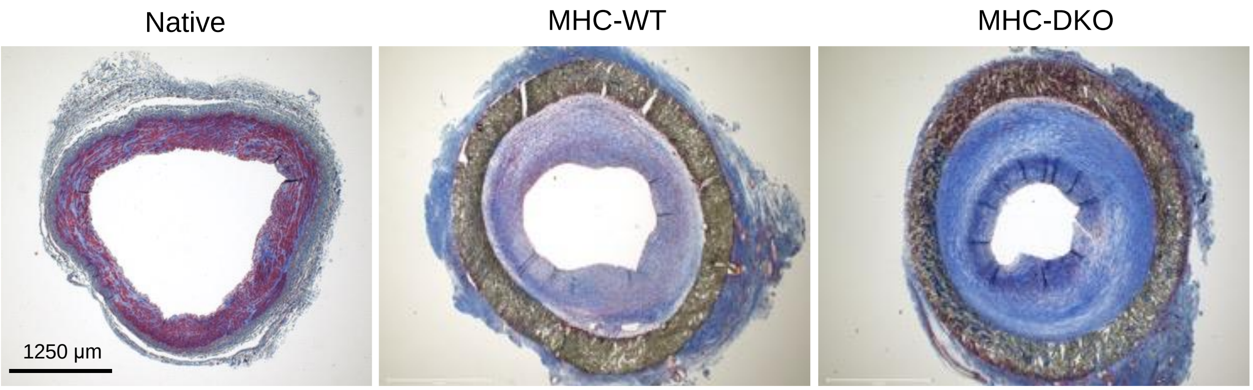

Figure S9. Trichrome staining. Related to Figure 5.
Shown are representative images of trichrome staining from a native femoral artery vessel, MHC-WT graft and MHC-DKO graft. Trichrome stains collagen in blue and smooth muscle cells (SMCs) in red.

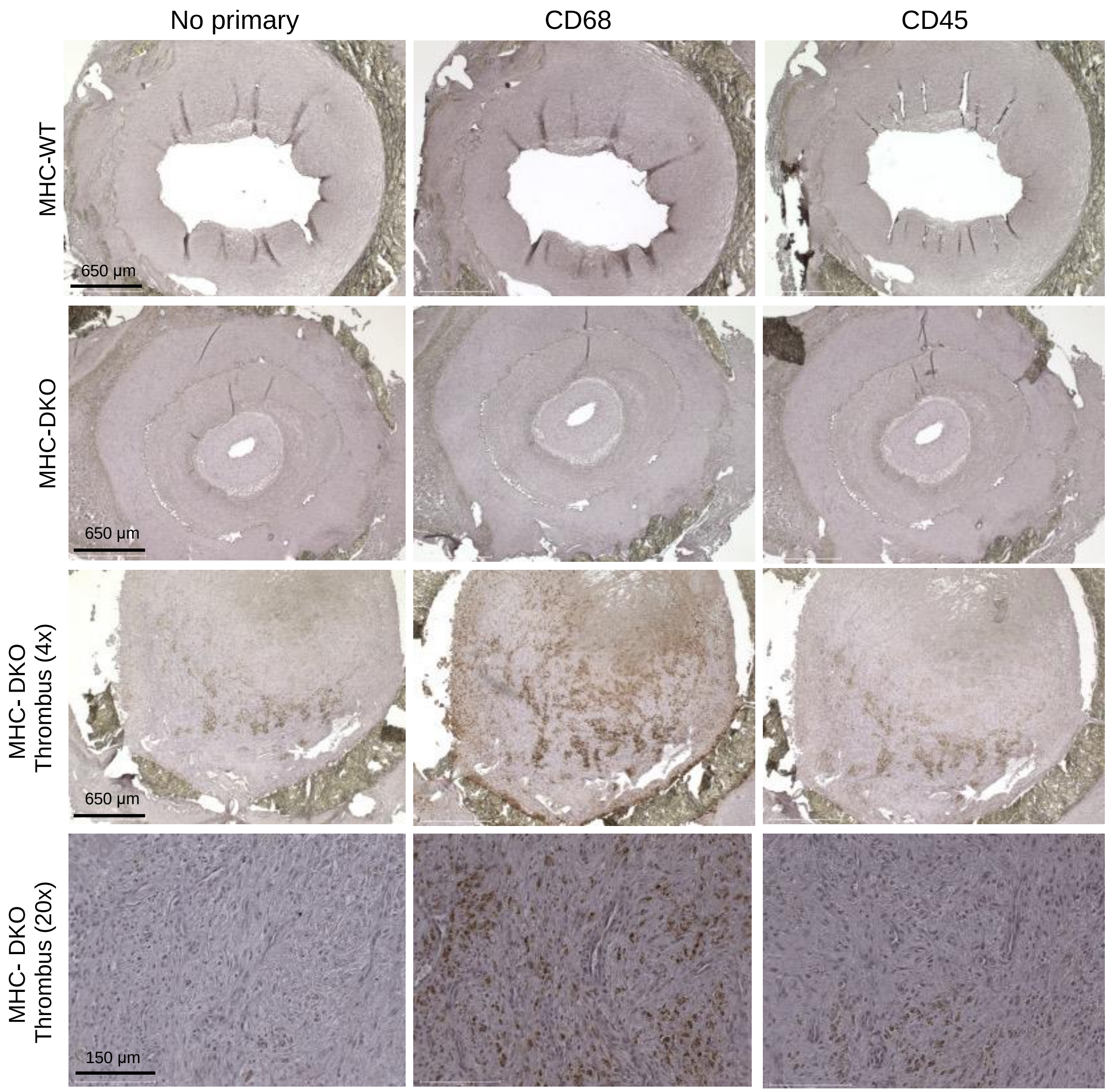

Figure S10. Immunohistochemical staining for macrophages and leukocytes. Related to Figure 5.
Shown are representative images from MHC-WT, MHC-DKO, and MHC-DKO with thrombosis grafts stained for CD68 (macrophages) and CD45 (leukocytes).

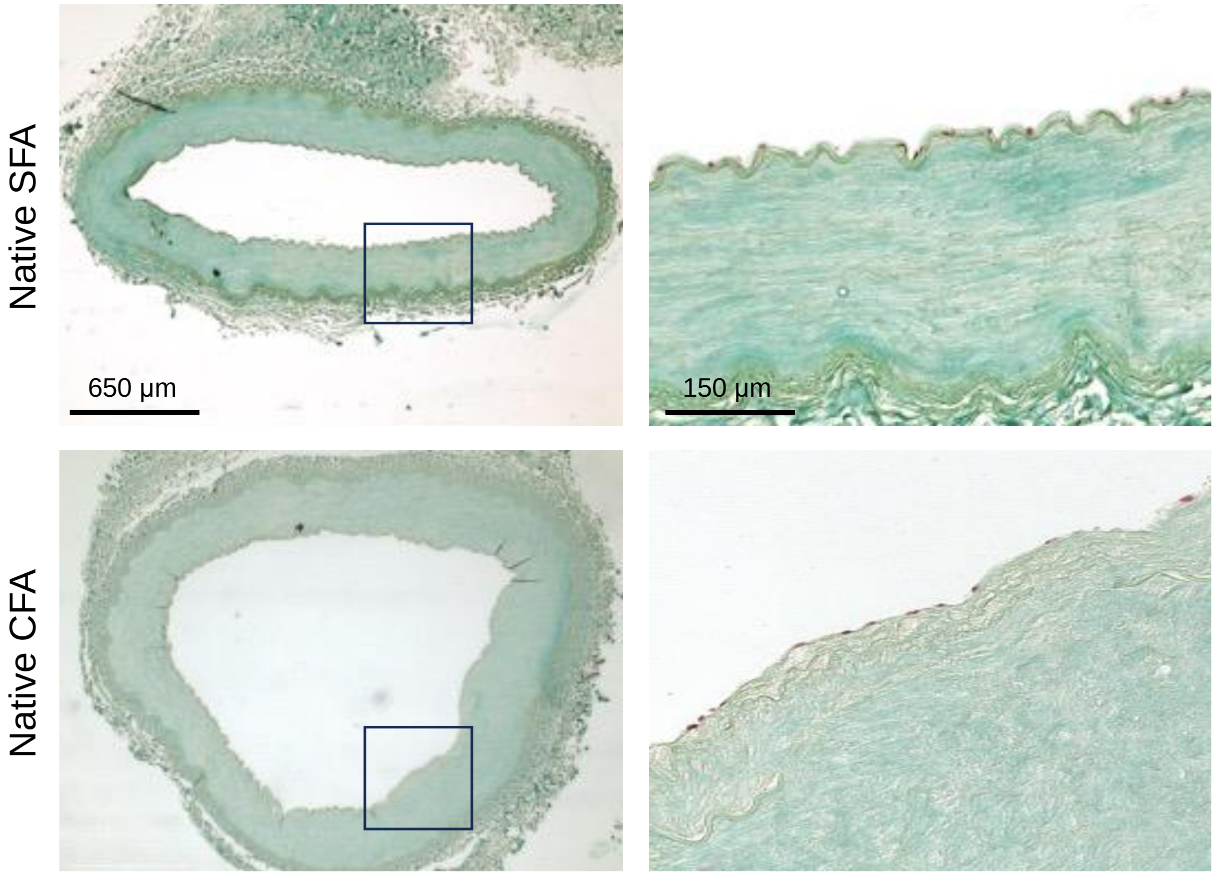

Figure S11. ERG immunohistochemistry in native vessels. Related to Figure 6.
a, Immunostaining of ERG in native superficial femoral artery and common femoral artery showing the presence of endothelium. ERG positive cells are red.

Table S1. Compound Structures of Small Molecules. Related to Figure 1.

Compound ID	Compound name	Vendor/Library	Catalog	Concentration	Smiles/Stucture
C21	Z220377028	EnamineStore	T6275935	10 μM	ClC1=CC2=C(C=C1)C(=CC=N2)N1CCN(CC1)S(=O)(=O)N1CCCCCC1
C23	Z64462386	EnamineStore	T6051787	3 μM	CCC1=CC=C(C=C1)S(=O)(=O)N1CCCC2=C1C=CC(C)=C2
C33	1-Octadecyl-2-methylglycero-3 PC	ENZO Bioactive lipid	L-108	2 μM	CCCCCCCCCCCCCCCCCCOC[C@H](COP([O-])(=O)OCC[N+](C)(C)C)OC
8510	Z126978510	EnamineStore	Z12697851(	2-5 μM	https://enaminestore.com/catalog/Z126978510

Table S2. MHC Haplotypes Donor Rhesus ESC Line and Graft Recipient Animals. Related to Figure 4.

Id	Implant Group	Concatenated Haplotypes
Rh8021	ePTFE Rh420 WT	A224.02, A004.01, B001.01, B012.02/03
Rh2499	ePTFE Rh420 WT	A004.01, A023.01, B069.02, B047.01, DR04.01, DR06.01
Rh2904	ePTFE Rh420 WT	A001.01, A018.02, B048.01, B013.02, DR16.01, DR02.01'
Rh2915	ePTFE Rh420 WT	A001.01, A006.01, B012.02/03, B001.01, DR09.01, DR06.01
Rh2916	ePTFE Rh420 WT	A002.01, A004.01, B001.01, B015.01, DR16.01, DR09.01
Rh2908	ePTFE Rh420 WT	A012.01, A002.01, B001.01, B069.01, DR06.01, DR11.01
Rh2910	ePTFE Rh420 DKO	A008.01, A023.01, B001.01, B012.02/03, DR16.01, DR11.01
Rh2912	ePTFE Rh420 DKO	A028.01, A019.01, B017.01, B001.01, DR04.01, DR04.01
Rh2913	ePTFE Rh420 DKO	A001.01, A007.01, B045.01, B047.01, DR14.01, DR16.01
r05041	ePTFE Rh420 DKO	A004.01, A008.01, B012.01, B069.01
Rh2917	ePTFE Rh420 DKO	A002.01, A002.01, B001.01, B012.01, DR16.01, DR03.06
Rh2914	ePTFE Rh420 DKO	A028.01, A006.01, B017.01, B001.01, DR04.01, DR06.01
r05089	ePTFE Rh420 naked aspirin matched	A002.01, A023.01, B001.01, B056.01, DR04.01, DR03.06
r06031	ePTFE Rh420 naked aspirin matched	A012.01, A006.01, B012.02/03, B048.01, DR15.01/02, DR01.01
r06035	ePTFE Rh420 naked aspirin matched	A001.01, A001.01, B048.01, B055.01, DR03.07, DR16.01
r11074	ePTFE Rh420 naked aspirin matched	A004.01, A008.01, B012.02/03, B001.01, DR14.01, DR14.01
r13097	ePTFE Rh420 naked aspirin matched	A008.01, A004.01, B001.01, B001.01, DR04.01, DR06.01
rhbk86	ePTFE Rh420 naked aspirin matched	A002.01, A004.01, B080.01, B015.01, DR03.01, DR03.01
r13005	ePTFE Rh420 WT VTN coating	A001.01, A008.01, B001.01, B017.01
rh2500	ePTFE Rh420 WT VTN coating	NA
r05045	ePTFE Rh420 WT VTN coating	A004.01, A011.01, B043.01, B017.01, DR03.01, DR13.01
rh2833	ePTFE Rh420 WT VTN coating	A019.01, A004.01, B001.01, B002.01, DR_unk, DR14.02
rh2837	ePTFE Rh420 WT VTN coating	A023.01, A026.01, B012.02/03, B012.02/03, DR04.01, DR09.01
r14100	ePTFE Rh420 WT VTN coating	A004.01, A002.01, B055.01, B012.01, DR04.01, DR16.01
Rh420 ESC	Donor AEC's	A001, A008, B015b, B029, DR03f, DR15a/b

Table S3. Media. Related to STAR Methods.

	Medium components	E8	E12	E8BAC	E8BAC2	Five factors	FVIR	Vendor	Cat#
DF3S	DMEM/F12	+	+	+	+	+	+	Thermo Fisher	Customized order
	L-ascorbic acid-2-phosphate magnesium (64 ng/ml)	+	+	+	+	+	+		
	Sodium selenium (14 ng/ml)	+	+	+	+	+	+		
	$NaHCO_3$ (543 µg/ml)	+	+	+	+	+	+		
	Transferrin (10.7 µg/ml)	+	+	+	+	+	+	Fisher Scientific	2914-HT-001G
	Insulin (20 µg/ml)	+	+	+	+		+	Sigma	I9287-5ML
	FGF2 (100 ng/ml)	+	+	+	+	+	+	Homemade	
	TGFβ1 (1.7 ng/ml)	+	+	+	+			R&D Systems	240-B
	BMP4 (5 ng/ml)			+	+			R&D Systems	314-BP
	Activin A (25 ng/ml)			+	+			R&D Systems	338-AC
	CHIR99021			**1 µM**	**2 µM**			R&D Systems	4423
	VEGFA165 (50 ng/ml)					+	+	R&D Systems	293-VE
	SB431542 (10 µM)					+	+	R&D Systems	1614
	RESV (5 µM)					+	+	R&D Systems	1418
	L690 (10 µM)					+		R&D Systems	0681
	GlutaMAX		**1x**					Life Technologies	82043745
	Chemically Defined Lipids		**1x**					Thermo Fisher	11905031
	rhNodal (50 ng/ml)		+					R&D Systems	3218-ND
	Glutathione (1.94 µg/mL)		+					Sigma	G4251

Table S4. gRNAs, Primers and Probes. Related to STAR Methods.

Human	CDH5 gRNA1	CCGGGAGGAGCTGCTGTATT	CDH5 -PuroR gRNA
	CDH5 gRNA2	AGGTCACTCTGGGCCTGGGG	CDH5 -PuroR gRNA
	J531	CAGGGCATCCCAGCTTTACT	CDH5 -PuroR ,Genotyping, 5' Arm 1091
	J532	GTACTCGGTCATAGGTCCAGG	
	J533	AGCGCATCGCCTTCTATCG	CDH5 -PuroR ,Genotyping, 3' Arm 1390
	J534	GCCACTTCTCCAAGGTGTGT	
Rhesus	PECAM1 gRNA1	CGCTGTCTAAGTTCCATCAA	gRNA for PECAM1-Puro -PGKNeo
	PECAM1 gRNA2	TCCTTCCAGGGATGTGCATC	
	J523	TGGACCATCTAAGTTCAGTCCT	PECAM1-PuroR, Genotyping 5' Arm, 988 bp
	J524	GAGGCCTTCCATCTGTTGCT	
	J525	ATCGCCTTCTTGACGAGTTCTT	PECAM1-PuroR, Genotyping, 3' Arm,1345bp
	J526	AGACCTCAGGAGACCACTTCT	
	B2M gRNA	GAGTAGCGCCAGCACGGCTA	rhesus B2M gRNA
	B2M Set 3 F	CCAGTTTGCAATGCGAGGAG	For rhesus B2M-/- genotyping
	B2M Set 3 R	CTTTGGGATGAGCCTACCCG	
	CIITA gRNA	ATGGAGTTGGGGCCCCTAGA	rhesus CIITA gRNA
	CIITA F	CCACTCATGGCATTGCCAAATGTGG	For rhesus CIITA-/- genotyping
	CIITA R	GCTGAACTGGTCACAGTTGATGG	
	PuroR FWD Set 1	GTC ACC GAG CTG CAA GAA	Copy No. of PuroR for both human and rhesus
	PuroR REV Set 1	CCG ATC TCG GCG AAC AC	
	PuroR PRB Set 1	/56-FAM/TCG ACA TCG /ZEN/GCA AGG TGT GGG T/3IABkFQ/	
	TERT FWD Set 1	GAC CAA GCA CTT CCT CTA CTC	Copy No. control for human
	TERT REV Set 1	GGA ACC CAG AAA GAT GGT CTC	
	TERT PRB Set 1	/56-FAM/AGA GAG CTG /ZEN/AGT AGG AAG GAG GGC /3IABkFQ/	
	HR4 FWD Set 3	GTGTGGGCTAAGATGGCATAA	Copy No. control for both human and rhesus
	HR4 REV Set 3	CTCCATTCCGAAAGAGCTTCA	
	HR4 PRB Set 3	/56-FAM/AGGGATAAT/ZEN/CGGATTTCTCCGTCATGA/3IABkFQ/	
